# Research Progress of MEMS Gas Sensors: A Comprehensive Review of Sensing Materials

**DOI:** 10.3390/s24248125

**Published:** 2024-12-19

**Authors:** Yingjun Wu, Ming Lei, Xiaohong Xia

**Affiliations:** 1 Ministry-of-Education Key Laboratory for the Green Preparation and Application of Functional Materials, School of Materials Science & Engineering, Hubei University, Wuhan 430062, China; 202231113021007@stu.hubu.edu.cn; 2 Wuhan Micro & Nano Sensor Technology Co., Ltd., Xingye Building, Wuda Science and Technology Park, Donghu New Technology Development Zone, Wuhan 430223, China; mnst@mnsenstech.com

**Keywords:** MEMS gas sensor, metal oxide semiconductor, carbon material, MOF, SAW

## Abstract

The MEMS gas sensor is one of the most promising gas sensors nowadays due to its advantage of small size, low power consumption, and easy integration. It has been widely applied in energy components, portable devices, smart living, etc. The performance of the gas sensor is largely determined by the sensing materials, as well as the fabrication methods. In this review, recent research progress on H_2_, CO, NO_2_, H_2_S, and NH_3_ MEMS sensors is surveyed, and sensing materials such as metal oxide semiconductors, organic materials, and carbon materials, modification methods like construction of heterostructures, doping, and surface modification of noble metals, and fabrication methods including chemical vapor deposition (CVD), sputtering deposition (SD), etc., are summarized. The effect of materials and technology on the performance of the MEMS gas sensors are compared.

## 1. Introduction

With the development of industry and technology, the potential danger of toxic or flammable and explosive gases leaking in the production and transportation process needs to be monitored and brought to attention as early as possible. The advance of high-performance, low-cost gas sensors is one of the most effective solutions. Great efforts have been put into the investigation of novel materials and facile processes with the aim of improving response sensitivity, reducing reaction temperatures, and minimizing device sizes. MEMS sensors are sensors based on microelectromechanical systems, usually with small sizes (1 μm and 1 mm) and unique manufacturing methods. They have the advantages of miniaturization, low power consumption, easily configurable multifunctionality, and easy integration. Various MEMS sensors, including pressure, acceleration, gyro, flow, gas, and temperature sensors, have been developed. The MEMS gas sensor is one of the most frequently used gas sensors at present. Compared with other types of gas sensors, MEMS gas sensors can be combined with temperature and humidity sensors more easily, and gas-sensing arrays or grids can also be easily constructed. Additionally, they have high sensitivity, fast response, lower power consumption, and a smaller size, enabling increased practicality in a wider range of applications. The detection of different gases can be realized through the development and improvement of sensitive materials with different properties [1].

The performance of the sensor can be evaluated according to the sensitivity, resolution, detection limit (LOD), response time, recovery time, and long-term stability. In this paper, recent progress on MEMS gas sensors including hydrogen, carbon monoxide, nitrogen dioxide, hydrogen sulfide, and ammonia sensors are reviewed in detail, and the above performance parameters of the gas sensor with different materials are compared. Firstly, we discuss the properties of different materials used in MEMS gas sensors. Then, the properties, application areas, and research progress regarding the five commonly used gas sensors are described in detail. Finally, we provide a summary of the performance of the various MEMS gas sensors, the remaining problems, and possible solutions to solve these problems in the future.

## 2. Materials for MEMS Gas Sensors

**Figure 1:**
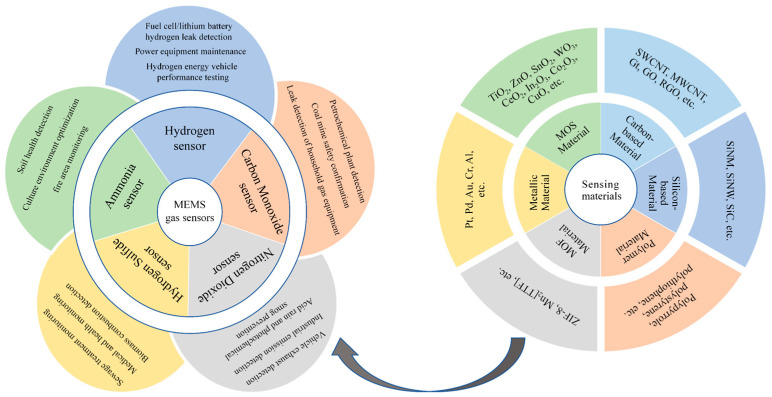
Summary of sensing materials and application areas for five MEMS gas sensors.

In recent years, research on different types of sensing materials for MEMS gas sensors has continued to expand, containing metal materials, metal oxide semiconductors (MOSs), metal–organic frameworks (MOFs), graphene and its derivatives, carbon nanotubes and their derivatives, nano-metal particles, transition metal dihalides (TMDs), etc. (Figure 1) [2,3]. Among them, MOS material is the most widely used MEMS gas-sensitive material because of its excellent performance and outstanding stability.

### 2.1. Metallic Material

Metal materials, including Pt, Pd, Au, Cr, and Al, are used in MEMS gas sensors in the form of metal nanoparticles, metal core–shell structures, and metal films. They meet the needs of MEMS sensing materials for suitable surface area, high diffusion rate, fast adsorption/desorption kinetics, and monitoring significant changes in material properties in the presence of gas molecules. In addition, alloys, metallic glass (MG), and other materials can be created through the combination of a variety of metal materials to achieve further improvement in performance.

### 2.2. Metal Oxide Semiconductor Materials

MOSs are the most widely used MEMS gas-sensing materials because of their excellent performance and good chemical and physical stability. The relationship between the resistance value of an MOS and the gas concentration on its surface is used to quantize the gas response. MOS-based materials can be divided into two types: N-type materials like TiO_2_, ZnO, SnO_2_, WO_3_, CeO_2_, In_2_O_3_, etc., and P-type materials such as Co_2_O_3_, CuO, and so on [4]. The difference between the two is that the charge carriers in N-type materials are electrons, while the charge carriers in P-type materials are holes [5]. The concentration of holes in P-type materials and electrons in N-type materials varies in different gas environments [5]. The relationship between the resistance value and gas concentration in an MOS gas sensor is related to its sensitivity characteristics. When an N-type MOS gas-sensitive material is exposed to air and heated to several hundred degrees Celsius by the heating wire, the oxygen in the air reacts at high temperature, trapping the electrons in the gas-sensitive material to generate active adsorbed oxygen negative ions (O^2−^, O^−^, and O_2_^−^), as shown in reaction Formulas (1)–(3):

O_2(gas)_ ↔ O_2(ads)_ (1)

O_2(ads)_ + e^−^ = O_2_^−^_(ads)_ (2)

O_2_^−^ + e^−^ = 2O^−^_(ads)_ (3)

The oxygen species adsorb on the gas-sensitive material, attract free electrons, and reduce the flowing electrons in the semiconductor material, thereby increasing the sensor resistance. When the reducing gases, such as H_2_ and CO, appear, the reducing gases interact with the adsorbed negative oxygen ions, and the originally adsorbed electrons are released into the gas-sensitive material, which increases the sensor current and decreases the resistance [5]. With the increase in the reducing gas concentration, the resistance value of the sensor decreases gradually. Similarly, if the test gas is oxidizing, the resistance value will increase with the increase in concentration [5].

When a P-type semiconductor is exposed to air, oxygen molecules capture the electrons inside the gas-sensitive material and produce oxygen negative ions (O^−^), forming a hole accumulation layer (HAL) on its surface, resulting in a reduction in the resistance of the P-type semiconductor material [5]. When reducing gas appears, oxygen anions react with it, the surface cavity accumulation layer becomes thinner, and the resistance value increases. On the contrary, when oxidizing gas is present, the resistance value decreases as its concentration increases.

Doping, noble metal modification, and heterostructure construction have been proposed to improve the gas-sensitive properties of MOS-based materials [4]. Doping refers to the introduction of heteroatoms into the MOS lattice, and it is a recognized as an effective way to change the sensing properties of materials, in which one or more metal atoms can be introduced. Structure defects and surface oxygen vacancies can be created through doping, thus promoting surface reactions and improving the gas-sensitive properties of materials [4]. Noble metal particles have electron sensitization and chemical catalysis; modification of MOS materials with noble metal particles can also improve their sensing performance [4]. The construction of heterogeneous structures refers to the realization of p-n, p-p, and n-n heterogeneous structures by the combination of two or more metal oxide semiconductor materials [5]. The heterojunction formed between the two MOS materials can often increase the mobility of charge carriers, thus increasing the gas-sensing response [4]. The heterogeneous materials are usually prepared by the hydrothermal method, electrospinning method, solvothermal method, chemical vapor deposition method, physical vapor deposition method, radio frequency sputtering method, etc. Sometimes it may be necessary to choose two or more ways to prepare materials to achieve changes in composition or structure to obtain better properties [6].

### 2.3. Carbon-Based Material

The commonly used carbon-based materials (CBMs) for MEMS gas sensors are graphene and carbon nanotubes (CNTs). Both single-walled carbon nanotubes (SWCNTs) and multi-walled carbon nanotubes (MWCNTs) have the potential to create high-performance gas sensors for the detection of a range of toxic gases, especially in the field of intelligent sensing and monitoring of large power equipment faults such as oil-immersed transformers [7]. However, these sensors still have the problem of the amount of detected gas being limited and the sensitivity needing to be further improved. Materials related to graphene include graphite sheets (Gt), graphene oxide (GO), reduced graphene oxide (RGO), etc. A GO sheet is the product of chemical oxidation and stripping of graphite powder. RGO is a two-dimensional layered carbonaceous material with a large number of active functional groups on its surface, which makes the sensing material more sensitive to gas molecules.

### 2.4. Silicon-Based Material

Silicon-based materials (SBMs), including silicon nanomembranes (SiNMs), silicon nanowires (SiNWs), and SiC, are also used in MEMS gas sensor preparation. Among them, SiNMs have a variety of electrical and material characteristics and can be used to make flexible RF thin-film transistors, microwave switches, and solar cells [8]. The silicon-based material gas sensor has the characteristics of strong stability, high reliability, and non-ageing and can be applied to the production of gas sensors requiring mobile operation in the future.

### 2.5. Polymer Material

Polymer gas-sensitive materials such as polypyrrole (PPy), polystyrene (PS), polythiophene (PTh), and other polymer materials are often used to develop MEMS gas sensors. Gas sensors made of polymer materials often have high flexibility and sensitivity. Functionalization of organic active materials with special functional groups and the preparation of composite films that provide more active sites and interfaces are effective means to improve the selectivity and sensitivity of polymer gas sensors.

### 2.6. Metal–Organic Framework Material

MOFs are a new generation of porous materials with large porosity and pore size variations, very high surface area (up to 10,000 m^2^/g), low density, and chemical tunability, and they have been widely studied and applied in many fields, including gas storage and separation, catalysis, biomedicine (such as drug storage and delivery), etc., [6]. In recent years, MOF materials have also often been used in the preparation of MEMS gas sensors.

## 3. Hydrogen Sensors

Hydrogen is a highly anticipated new energy gas. Due to its low molecular weight, it can easily leak during production and transportation, and it can easily explode in case of open fire. Therefore, it is necessary to develop MEMS sensors with high H_2_ sensitivity and good selectivity. In the field of new energy, these sensors can be used to ensure the safe operation of lithium batteries and fuel cells, etc.; in the industrial field, they can be used to detect hydrogen leaks in the process of steel manufacturing, and in the field of environmental protection, they can be used to ensure the safety and reliability of hydrogen energy use. In recent years, researchers have continued to develop hydrogen sensors with better performance and lower operating temperatures. Table 1 lists the performance of some hydrogen sensors developed in recent years.

**Table 1:** Performance comparison of MEMS hydrogen gas sensors and sensing materials.

Sensitive Material Category	Sensitive Material	Fabrication Technique	Working Temperature (°C)	Detection Range (ppm)	Response Time (s)	Recovery Time (s)	Sensitivity	Ref.
Metal	Pt	Sputter technique	30–200	1000–10,000	N/a	N/a	3.4@1000 ppm ^d^	[9]
Metal	Pd	Polyimide-based microfabrication process	25–85	5000–40,000	<5	N/a	N/a	[10]
Alloy	Pd/Au	Direct current (DC) magnetron SD	60	5–30,000	22	160	3.3%@30,000 ppm ^c^	[11]
Alloy	Pd/Mg	DC magnetron sputtering	100	N/a	1	60	N/a	[12]
Alloy	Pd/Ni	E-beam evaporation	25	80–5000	84	96	7.1%@5000 ppm ^c^	[13]
MG	Pd_78_Cu_5_Si_17_	Multi-target sputtering	Room temperature	0.05–40,000	2.1	N/a	N/a	[14,15]
CBM	Pd/CNT	Electrochemical deposition process	Room temperature	30	3	N/a	75%@500 ppm ^c^	[16]
Metal/CBM	Pd cluster/graphene electrodes	Electrodeposition method	Room temperature	N/a	N/a	N/a	N/a	[17]
SBM	Pd/SiNM	E-beam evaporation	Room temperature	50–5000	22	N/a	764%@500 ppm ^c^	[7]
SBM	Pd/Si nanomesh	Nanosphere lithography	Room temperature	1000–10,000	5	13	27%@10,000 ppm ^c^	[18]
SBM	3C–SiC	CVD	Room temperature	2000–20,000	N/a	N/a	N/a	[19]
SBM	Pd/SiNW	E-beam evaporation	Room temperature	100–1000	N/a	N/a	154.5%@1000 ppm ^c^	[20]
MOS	SnO_2_ with defects	Drop sintered	250	0.1–6	7	12	2.3@6 ppm ^a^	[21]
MOS	WO_3_	Radio frequency (RF) magnetron method	20–350	5–1000	120–180	120–180	1300@1000 ppm ^a^	[22]
MOS	Ammonia plasma modification ZnO-NWs	Sputter technique	Room temperature	500–2500	N/a	N/a	27%@2500 ppm ^c^	[23]
Metal/MOS	Pd/ZnO-NRs	Sol–gel technology	Room temperature	0.2–1000	18.8	N/a	91%@1000 ppm ^c^	[24]
Metal/MOS	Pd nanotube/ZnO	Low-temperature, wet-chemical process/hydrothermal method	Room temperature	N/a	N/a	N/a	1500%@1000 ppm ^c^	[25]
Metal/MOS	Pt/TiO_2_	Calcining method	500–800	N/a	0.04 (H_2_) 0.02 (O_2_)	N/a	N/a	[26]
Metal/MOS	Pt/Nb/TiO_2_	Electrostatic microspray method	40	200–1000	31	270	12.3@1000 ppm ^a^	[27]
Metal/MOS	Pd/SnO_2_ thin films	Sol–gel method	75	150–1000	182	108.9	165@1000 ppm ^a^	[28]
MOS/MOS	MOF-derived WO_3_-C/In_2_O_3_	Drop coating	250	5–2000	1.9	9.2	10.11@1000 ppm ^a^	[29]
MOS/metal/MOF	ZnO/Pd/ZIF-8 nanowires	N/a	200	10–50	N/a	8.33	6.2@50 ppm ^b^	[30]

^a^ R = R_a_/R_g_. ^b^ R = R_g_/R_a_. ^c^ R = |R_g_ − R_a_|/R_a_ × 100%. ^d^ R = |R_g_ − R_a_|/R_g_ × 100%.

### 3.1. Metallic Material

#### 3.1.1. Metal

**Figure 2:**
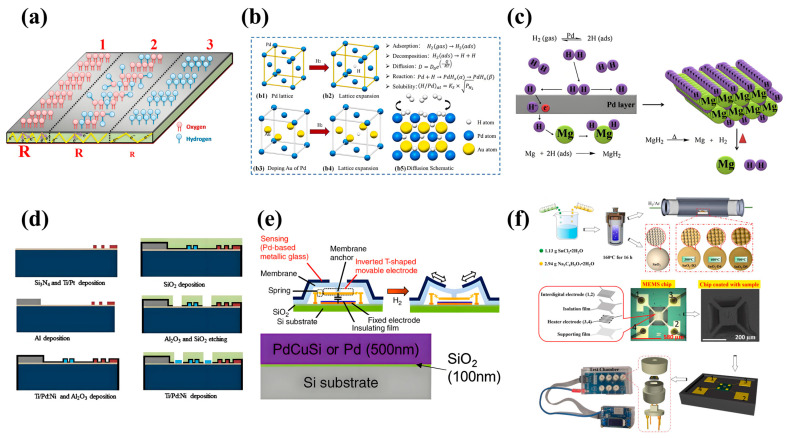
(**a**) Schematic diagram for H_2_ ad/absorption on the surface of Pt film [9]. Copyright 2016, Elsevier. (**b**) Schematic diagram for hydrogen-sensitive mechanism on Pd alloy thin film [11]. Copyright 2023, Elsevier. (**c**) Schematic diagram of hydrogenation and dehydrogenation of Pd/Mg thin film [12]. Copyright 2015, Elsevier. (**d**) Fabrication procedure of the hydrogen sensor array combined with temperature sensor and planar microheater [13]. Copyright 2021, Elsevier. (**e**) Schematic cross-sectional diagrams of MEMS capacitive H_2_ sensor and sensing mechanism and sample structure for analysis [14]. Copyright 2018, IEEJ. (**f**) Schematic of the SnO_2_-D MEMS sensor formation process and production principle [21]. Copyright 2022, Elsevier.

Sennik et al. used RF sputtering to develop an MEMS H_2_ sensor using a Pt film deposited on a glass substrate as the sensing material [9]. The induction mechanism is shown in Figure 2a: After exposure to hydrogen, the surface oxygen atoms of Pt film will be replaced by hydrogen atoms, without forming a bulk hydride phase [9]. And the replacement process is reversible [9]. Under dry airflow conditions, the optimal operating temperature of a 2 nm thick Pt film H_2_ sensor is 30 °C [9]. The H_2_ detection concentration range of the sensor is 0.1–1%, and the response value R_a_/R_g_ = 3.4 when the H_2_ concentration is 1000 ppm [9]. Walewyns et al. introduce a Pd/Al-based 3D-MEMS capacitive H_2_ sensor. The Pd hydrogenation reaction gives it ultra-low power, a fast response (5 s), high sensitivity to the lower explosive limit (LEL), and high selectivity for hydrogen, which has higher safety in future low-power sensing applications with high selectivity and dynamics [10].

#### 3.1.2. Alloy

Gong et al. reported an MEMS resistive H_2_ sensor based on a Pd-Au alloy thin film produced by DC magnetron SD [11]. Figure 2b illustrates the lattice expansion and diffusion properties of Pd-based alloy films [11]. After the hydrogen atom reacts with the Pd atom to form PdH_x_, lattice expansion occurs, and the expanded lattice increases the movement space and diffusion rate of the hydrogen atom in the alloy film, thus improving the response speed of the sensor [11]. The state of hydrogen atoms adsorbed and diffused into Pd-based films is affected by the partial pressure of hydrogen in the environment [11]. When the hydrogen partial pressure is stable, the physical and chemical properties of the film also remain stable [11]. The sensor operates at 60 °C, with a detection range of 5 ppm to 3% H_2_ and a response value of 3.3% when the hydrogen concentration is 3% [11]. The response time and recovery time are 22 s and 160 s, respectively [11]. The sensor has low power consumption, high production, high performance, good repeatability, long-term stability, and high selectivity for H_2_ [11]. Sanger et al. developed a Pd-coated Mg film deposited on an electrochemically etched porous silicon substrate using DC magnetron sputtering technology and used the material for the development of MEMS H_2_ sensing [12]. Figure 2c illustrates the reaction process of Pd/Mg films during hydrogenation and dehydrogenation [12]. The formation of MgH_2_ in the Mg layer leads to a change in the resistance of the Pd/Mg layer [12]. The porous structure of the silicon substrate allows hydrophobic and high-surface-area film deposition and prevents the material from delaminating from the surface under humid conditions [12]. The resistance of Pd/Mg films is reversible and mechanically stable during multiple hydrogenation/dehydrogenation cycles [12]. The Pd/Mg thin-film H_2_ sensor has the advantages of fast response (1 s), economy, and sensitivity at low temperatures [12]. Kondalkar et al. reported a Pd-Ni (3%) based bendable resistance MEMS H_2_ sensor for dissolved hydrogen analysis (DHGA) [13]. The manufacturing steps are shown in Figure 2d, including the deposition of Si_3_N_4_ and SiO_2_ by PECVD, the deposition of Ti/Pt/Ni by electron beam evaporator, the deposition of an aluminum wire, and the formation of Al_2_O_3_ by the atomic layer deposition process [13]. Atomic layer deposition (ALD) technology is also utilized to increase the surface area of the material to improve gas-sensitive properties [13]. It has a good response to 80–5000 ppm H_2_ concentration and has the highest response value at 25 °C with a response value (∆R/R_0_) of 7.1% at 5000 ppm [13]. The research results show that the developed sensor has stable performance and is very promising in the application of transformer monitoring [13].

#### 3.1.3. Metallic Glass

Yamazaki et al. fabricated a capacitive MEMS H_2_ sensor based on Pd-based metallic glass (MG) by multi-target sputtering [14,15]. As shown in Figure 2e, a system with two DC sources and one RF source is employed for sputtering Pd, Si, and Cu [14]. The sputtering system is capable of controlling the compositional ratio through the modification of the sputtering intensity for both DC and RF sources, thereby elucidating the ease and precision inherent in RF sputtering processes [14]. The research shows that PdCuSi, as a PD-based MG, has a good application prospect in capacitive MEMS H_2_ sensors [14]. In addition, several PdCuSi compositions with different component contents were compared. Only Pd_78_Cu_5_Si_17_ was an amorphous metal with MG characteristics [14]. Under the condition of a low hydrogen concentration of 0.05 vol% to 4.0 vol%, its strain well followed Sieferts’s law, indicating that hydrogen exists in MG in a diffused state [15]. The sensor is manufactured by surface multi-target sputtering technology and has the characteristics of non-hysteresis and fast response (2.1 s) at room temperature, so it has obvious advantages in hydrogen leak detection applications [14].

### 3.2. MOS Material

#### 3.2.1. MOS

**Figure 3:**
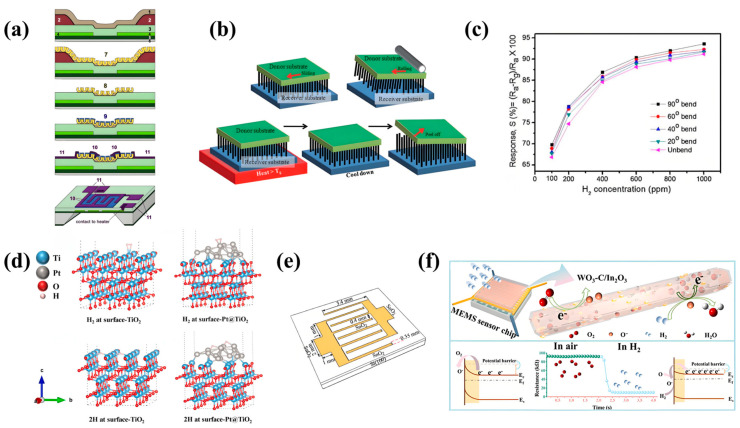
(**a**) Fabrication schematic diagram of porous anodized alumina-loaded WO_3_ sensing layer based on micro-hot plate [22]. Copyright 2013, Elsevier. (**b**) Sliding transfer method, rolling transfer method, and heat transfer method schematic diagram of ZnO nanowire MEMS H_2_ sensor [23]. Copyright 2011, Royal Society of Chemistry. (**c**) The response of Pd/ZnO hydrogen sensors with different bending angles varying with H_2_ concentrations [24]. Copyright 2013, Elsevier. (**d**) Schematic atomic structure of adsorption of H_2_ by pure TiO_2_ and Pt/TiO_2_ and separation of H on pure TiO_2_ and Pt/TiO_2_ [27]. Copyright 2024, Elsevier. (**e**) Pd grid contact diagram deposited on SnO_2_ nanocrystalline film [28]. Copyright 2016, Springer Nature. (**f**) Schematic view of H_2_ sensing mechanism for WO_3_-C/In_2_O_3_ sensor [29]. Copyright 2024, Elsevier.

A flexible H_2_ sensor also has good mechanical bending test results and can be applied to vehicles, aircraft, aviation, and portable electronic devices in the future [24]. Luo et al. synthesized SnO_2_ materials with oxygen vacancy defects used for MEMS H_2_ sensors [21]. As shown in Figure 2f, the flexible hydrothermal method was used to prepare ZnO nano-Petri dishes with oxygen-rich vacancies. Samples (SnO_2_-D3, SnO_2_-D4, SnO_2_-D5, SnO_2_-D6, and SnO_2_-D7) were obtained by annealing SnO_2_ at different temperatures (300 °C, 400 °C, 500 °C, 600 °C, and 700 °C), and the sample drops were coated on Pt digital electrodes, dried, and then sintered to complete the preparation [21]. When the SnO_2_-D sample is exposed in H_2_, lattice oxygen reacts with H_2_, forming an oxygen vacancy defect on the surface. The gas molecules react with chemisorbed oxygen, resulting in the release of more electrons after the oxidation–reduction reaction, making SnO_2_-D more sensitive and faster than the original SnO_2_ for gas sensing [21]. The original SnO_2_ has a response time/recovery time of 12 s/15 s for 6 ppm H_2_ at 250 °C, while the SnO_2_-D3, SnO_2_-D4, and SnO_2_-D5 sensors have a response time/recovery time of ~7 s/12 s under the same conditions [21]. Using XPS characterization, the results showed that the oxygen vacancy content of SnO_2_, SnO_2_-D_3_, SnO_2_-D_4_, and SnO_2_-D_5_ was 21.11%, 22.32%, 29.47%, and 23.65%, respectively [21]. Because SnO_2_-D4 had the highest oxygen vacancy content, its performance was also the best. When the relative humidity is 40%, the resistance values of SnO_2_, SnO_2_-D3, SnO_2_-D4, and SnO_2_-D5 to 3 ppm H_2_ are1.25 MΩ, 1.56 MΩ, 1.90 MΩ, and 1.45 MΩ, respectively [21]. Even with the lower specific surface area of SnO_2_-D4, gas-sensitive tests show that the SnO_2_-D4 MEMS sensor has a superior H_2_ response (R_a_/R_g_ = 2.3@6 ppm) and a very low LOD (0.1 ppm) due to the higher oxygen vacancy on the surface of SnO_2_-D4 [21]. The sensor is expected to be widely used in elastic catalysis, photocatalysts, and other fields [21]. Mozalev et al. prepared a practical MEMS H_2_ sensor with a fast response (2–3 min) and high sensitivity (R_a_/R_g_ = 1300@1000 ppm) using a nanostructured pore anodized aluminum oxide template WO_3_ layer [22]. The preparation process is shown in Figure 3a, including forming the Si_3_N_4_ layer on the silicon wafer, adding the polysilicon heater and SiO_2_ isolation layer to prepare the photoresist mask, sputtering the Al/Ti double layer on the photoresist mask to form porous anodic alumina (PAA) film, expanding the Al_2_O_3_ nanopore, and depositing the WO_3_ layer on the PAA film., annealing in the layer of SiO_2_ and forming a contact opening towards the heating end, forming a staggered electrode and an electrode contact pad, and etching the back wafer side to complete the gas-sensitive film [22]. The sensor developed can achieve low cost, low power consumption, and high capacity, which is conducive to the economic and environmental protection of hydrogen-based energy [22]. Ong et al. prepared a p-n junction diode-based ZnO nanowire MEMS H_2_ sensor by ammonia plasma modification [23]. ZnO nanowires are synthesized by the low-temperature hydrothermal method and transferred to a vinyl terephthalate (PET) substrate [23]. Three methods used in this process are shown in Figure 3b (slide transfer method, roll transfer method, and heat transfer method) [23]. The results show that the conductivity of ZnO nanowires synthesized by hydrothermal synthesis can be effectively adjusted by surface modification without heat treatment by using ammonia plasma, which guarantees the stability of the sensor at low temperatures [23]. At room temperature, when the H_2_ concentration is 2500 ppm, the sensor response value ∆R/R_0_ is 27% [23].

#### 3.2.2. Metal/MOS

Rashid et al. reported that a metal nanotube array was synthesized by a new low-temperature wet chemical process, and the tubular Pd nanostructure was directly formed on the sensor device by in situ dissolution of the ZnO nanowire template grown on the electrode surface by hydrothermal growth [24]. Figure 3c displays that under different bending conditions, the response value of the flexible sensor changes with the concentration of H_2_ [24]. Due to the higher surface reactivity in the bending state, the sensor’s response value is improved (∆R/R_0_ = 93.1%@1000 ppm, at 90° bend), which can accept more H_2_ molecules for active adsorption [24]. Zhang et al. developed a Pt/TiO_2_ sensor, and the addition of Pt improved the response rate of the TiO_2_ sensor when exposed to H_2_/O_2_ [26]. The surface of the sensing layer was modified with a solution containing H_2_PtCl_6_ and treated at different temperatures and times [26]. The results show that the response time of the sensor exposed to H_2_ and O_2_ for 2 h at 900 °C is 40 ms and 20 ms at the operating temperature of 500–800 °C [26]. Experimental results show that sensors with more platinum particles dispersed on titanium dioxide exhibit higher response values at low temperatures [26]. Zhang et al. produced an MEMS H_2_ sensor based on a Pt-modified Nb-doped TiO_2_ sheet, aiming to solve the challenge of MEMS sensors working in room-temperature and low-oxygen environments [27]. As can be seen from Figure 3d, when hydrogen is adsorbed on a pure (001) TiO_2_ plate, H_2_ molecules are more inclined to be adsorbed and stay at the active site of Ti (4) [27]. After hydrogen is adsorbed in the heterogeneous structure of Pt/TiO_2_, it will be catalyzed by Pt to split into atom H, and the split atom H tends to bind with Ti (4), which makes the sensor respond faster and the performance more stable [27]. The sensor can be used to detect hydrogen released during the charge and discharge of lithium-ion batteries (LIBs), addressing potential safety risks [27]. The Pt/TiO_2_ sensor achieves the advantages of compact size (0.05 cm^3^), low power consumption (0.1 mW at room temperature), excellent sensing performance, and easy integration [27]. The 90% RH response value can reach 12.3, and the sensor’s response and recovery time can reach 31 s/270 s at room temperature (40 °C) and 1000 ppm H_2_. On the surface of Pt/TiO_2_, H is more inclined to combine with Ti(4), which leads to a more stable structure [27]. Due to the diffusion of H atoms into the TiO_2_ lattice, the energy band structure of TiO_2_ changes, making the sensor have excellent sensing performance for hydrogen [27]. Kadhim et al. prepared high-quality nanocrystalline SnO_2_/Pd metal film MEMS H_2_ by the sol–gel method [28]. Figure 3e shows palladium mesh contacts deposited on nanocrystalline tin oxide films [28]. Metal–semiconductor–metal gas sensor components are manufactured by RF sputtering palladium grids on nanocrystalline tin oxide films [28]. The mask contains two electrodes, each consisting of four fingers, with a space of 0.4 mm between the two adjacent fingers and a width of 0.35 mm for each finger [28]. The nanocrystalline SnO_2_ film produced by adding glycerol has high porosity and good sensitivity, and the addition of Pd further improves the sensing performance of the sensor [28]. At an operating temperature of 75 °C, the sensor has a sensitivity of 165 for 1000 ppm H_2_ [28].

#### 3.2.3. MOS/MOS

Guo et al. synthesized MOF-derivative In_2_O_3_ by in situ coupling a carbon layer and WO_3_ and prepared an MEMS H_2_ sensor using the porous heterostructure WO_3_-C/In_2_O_3_ as a sensing material [29]. The self-assembly method is employed to make the c/h-In_2_O_3_ film composed of particles more compact, uniform, and continuous. The three-dimensional layered porous structure of WO_3_-C/In_2_O_3_ sensor material, which can promote H_2_ transmission and diffusion, is shown in Figure 3f [29]. The high sensitivity is mainly attributed to the high carrier mobility, low activation energy, and intrinsic noise brought by the C/MOS nanocomposites [29]. At the same temperature, when the mass ratio of WO_3_ to In_2_O_3_ is 9 wt% (WCI-9), compared with WCI-7 and WCI-11, the sensor has a higher response value (R_a_/R_g_ = 10.11@1000 ppm), and the sensor reaches the best working state at 250 °C [29]. The sensor has a low LOD for H_2_ (5 ppm), and the response value increases with increasing H_2_ concentration and remains stable over time. In addition, the WCI-9 sensor has a fast response/recovery speed of 1.9/9.2 s@220 ppm [29].

### 3.3. Carbon-Based Material

Weber et al. reported a high-performance flexible MEMS H_2_ sensor based on decorated Pd nanoparticles and single-wall carbon nanotubes [16]. The sensor is highly selective to H_2_, and the film responds best to H_2_ at 200 °C operating temperature [16]. In the range of H_2_ concentrations from 10 ppm to 50 ppm, the response value increases with the concentration increase. When the H_2_ concentration in the air is 0.05%, the response value reaches 75% [16]. After detection, T_res_ and T_rec_ are 3 s and 8.3 s, respectively [16]. Teleki et al. reported a flexible H_2_ sensor with an electrodeposition electrode based on a Pd-cluster-modified graphene electrode prepared by chemical vapor deposition [17]. The deposited palladium nanocluster (FPNC)–CG electrodes are sensitive to H_2_ at room temperature, and the performance will be improved with the increase in FPNC population [17].

### 3.4. Silicon-Based Material

**Figure 4:**
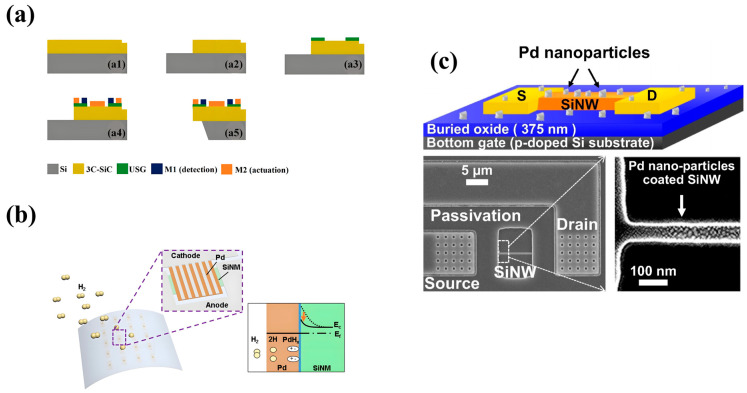
(**a**) Schematic diagram of the 5 lithographic steps of the process flow diagram of the 3C-SiC cantilever beam: (**a1**) patterning alignment crosses, (**a2**) defining cantilevers geometries, (**a3**) introduction of an isolation layer to separate 3C–SiC and metal contacts, (**a4**) metal deposition for electromagnetic actuation and inductive detection and (**a5**) cantilever releasing from the rear side, by etching the Si substrate with KOH [20]. Copyright 2024, Materials Science in Semiconductor Processing. (**b**) Energy bending diagram and sensing mechanism diagram of the flexible Pd/SiNM H_2_ sensor [7]. Copyright 2018, American Chemical Society. (**c**) Schematic of a SiNW FET with a bottom-gate structure for H_2_ sensing [19]. Copyright 2015, Solid-State Electronics.

Michaud et al. reported an MEMS H_2_ gas sensor based on a 3C-SiC microcantilever with a detection range of 0.2–2% [19]. The chemical adsorption of H_2_ molecules by the SiC layer causes a drastic change in the electronic properties of the SiC film [19]. This change is used to measure the concentration of H_2_ [19]. The 3C-SiC cantilever manufacturing process consists of five lithographic steps (Figure 4a): mode alignment intersections; define the cantilever geometry. An isolation layer is introduced to separate the contact between 3C-SiC and metal. For electromagnetic drive and induction detection of metal deposition, the cantilever is released from the rear by etching a silicon substrate with potassium hydroxide [19]. The sensor does not have a functionalized coating, which avoids a series of failure problems such as equipment aging, low reliability, and high response time that may be caused by the sensitive layer [19]. Cho et al. prepared a diode-type MEMS H_2_ sensor by releasing SiNMs from a rigid substrate, transferring them directly to a flexible substrate, and then performing metal deposition [7]. The flexible Pd/SiNM surface diagram and energy bend diagram reveal the sensing mechanism of H_2_ (Figure 4b) [7]. The cathode electrodes interlace; Pd and SiNMs form Schottky contact [7]. When the sensor is exposed to a H_2_ environment, the hydrogen atoms decomposed by H_2_ gas molecules diffuse into the Pd layer, forming PdH_x_ at the Pd/SiNM interface, and the Schottky barrier decreases [7]. The manufacturing process is simple and suitable for the chip level, with a wide detection range (50–5000 ppm), high sensitivity (∆R/R0 = 764%@500 ppm), and very low power (nW range) [7]. Choi et al. prepared a chemically gated transistor gas sensor based on SiNW, which is topped with a SnO_2_ film [20]. Figure 4c shows a SiNW FET schematic with a bottom gate structure for H_2_ sensing and scanning electron microscopy (SEM) images of Pd nanoparticles deposited on SiNW [20]. Here, SiNW is used as the bottom gate of the field-effect transistor. The sensor enables the decoupling of chemically sensitive areas from conductive channels to reduce drive voltage and improve reliability and battery supply capacity for applications in mobile and wearable sensor platforms [20]. They demonstrated the sensor’s operation at 1 V for mobile applications. The sensor can selectively detect H_2_, H_2_S, NO_2_, and other gases [20].

### 3.5. MOF Material

Weber et al. developed an MEMS H_2_ sensor based on a combination of Pd nanoparticle-modified ZnO nanowire and molecular sieve metal–organic skeleton nanomembrane (ZIF-8) [30]. Pd-NPs enable the sensor to achieve maximum signal response, while the ZIF-8 coating has excellent selectivity for H_2_, and ZIF-8/Pd/ZnO nanostructured materials significantly enhance the sensor sensitivity (R_g_/R_a_ = 6.2@50 ppm) [30].

## 4. Carbon Monoxide Sensors

Carbon monoxide is a typical toxic gas produced by incomplete combustion, and it can bind with hemoglobin and cause harm to human health. The working principle of CO sensors is usually based on chemical reactions such as electrocatalytic combustion or electrochemistry. The performance of some carbon monoxide sensors is shown in Table 2. MEMS CO sensors can be widely used in many fields, such as in the biomedical field to ensure human health, in the industrial field to monitor the petrochemical manufacturing process, and in the monitoring of atmospheric quality in the environment. Its good sensing performance is of key significance for safety and environmental protection applications, and the market size is also growing year by year. For example, Daly reported a new infrared MEMS sensor technology prepared by using a silicon-based, heat-isolated suspension bridge structure, which can be used for environmental monitoring of industrial pollutants (CO, CO_2_, NO_x_, etc.) [31].

The working mechanism of the CO sensor is based on the principle of a chemical reaction or physical reaction according to the different materials. The working principle of the chemical sensor is based on the oxidation reaction of CO with MOS, polymer, and other gas-sensitive materials. For example, when MOS material is exposed to CO, carbon monoxide molecules react with adsorbed oxygen ions on the surface of the material, as shown in reaction Formulas (4)–(6), electrons are re-released and migrate to the conduction band of the semiconductor material, and the energy band of the material is bent and the potential barrier is reduced, thus reducing the resistance [32].

O_2_^−^_(ads)_ + 2CO_(gas)_ = 2CO_2(gas)_ + e^−^ (4)

O^−^_(ads)_ + CO_(gas)_ = CO_2(gas)_ + e^−^ (5)

O^2−^_(ads)_ + CO_(gas)_ = CO_2(gas)_ + 2e^−^ (6)

A physical sensor performs measurements using the adsorption and desorption process of CO and a specific substance. Gas-sensitive materials such as MOS or CBM are used as adsorbents. When CO enters the sensor, it will be adsorbed on the surface of the adsorbent. When the concentration of CO increases, the concentration of CO on the adsorbent surface also increases. By measuring the change in CO concentration on the surface of the adsorbent, the CO concentration can be obtained indirectly.

**Table 2:** Performance comparison of carbon monoxide MEMS gas sensors and sensitive materials.

Sensitive Material Category	Sensitive Material	Fabrication Technique	Working Temperature (°C)	Detection Range (ppm)	Response Time (s)	Recovery Time (s)	Sensitivity	Ref.
MOS	TiO_2_ nanoparticle	Drop-coating method	500	1–75	30–60	550	N/a	[33]
MOS	Nanocrystalline SnO_2_	Sol–gel synthesis method	450	N/a	106	114	N/a	[34]
Metal/MOS	Al/TiO_2_ nanopowder	Combustion method	600	100–500	N/a	N/a	N/a	[35]
Metal/MOS	Pt/SnO_2_ nanoparticle	In situ deposition	350	8–50	N/a	N/a	N/a	[36]
Metal/MOS	Al/ZnO	Sol–gel technique	300	50	7	30	1.6@50 ppm ^b^	[37]
Metal/MOS	Ca/ZnO thin-film-coated langasite lanthanum gallium (LGS)	Spin coated	400	1000	87	132	2.469 kHz/ppm ^d^	[38]
CBM	SW-defect graphene	Drop coating	Room temperature	N/a	N/a	N/a	35.25% ^c^	[39]
Polymer	Ferrocene–chitosan	Drop-casting method	Room temperature	0–2000	38	64	108.85 Hz/ppm ^d^	[40]
Polymer	Cryptophane-A	Electrospray method	80	N/a	N/a	N/a	0.004 Hz/ppm ^d^	[41]
Metal/polymer	Fe-Al-doped PANI thin film	Vacuum deposition	Room temperature	10–150	5	10	800@150 ppm ^a^	[42]
Metal/polymer	PDPP4T-T-Pd (II)	Air–water interface coordination reactions of thymine groups with ions	Room temperature	0.01–100	N/a	N/a	N/a	[43]
Polymer/polymer	Poly (styrenesulfonate)/polyvinylpyrrolidone (PEDOT/PSS/PVP)	Traditional electrospinning	Room temperature	50	N/a	N/a	−54 Hz/ppm ^d^	[44]

^a^ R = R_a_/R_g_. ^b^ R = R_g_/R_a_. ^c^ R = |exp [(E′_g_ − E_g_)/2kT] − 1|. ^d^ Δf = 2f_0_^2^ Δm/A √ρ_q_μ_q_.

### 4.1. MOS Material

#### 4.1.1. MOS

**Figure 5:**
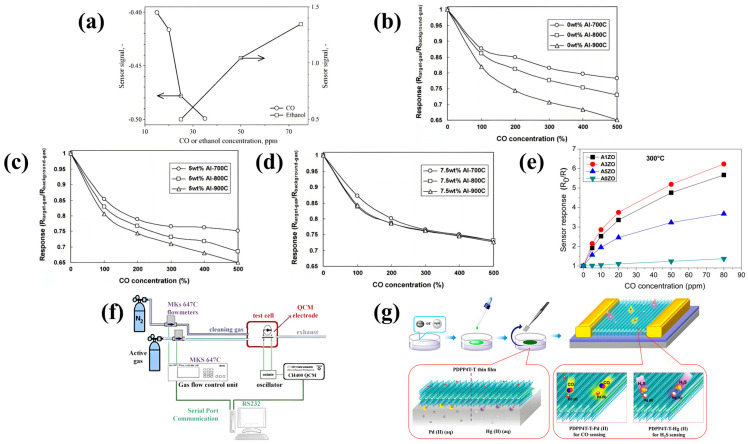
(**a**) Signal diagram of S2 sensor after heat treatment at 900 °C in a dry O_2_/N_2_ atmosphere at 500 °C, exposed to CO and ethanol [33]. Copyright 2006, Elsevier. (**b**) Diagram of the change in response value of 0 wt% Al-doped TiO_2_ sensor with CO concentration at 700 °C, 800 °C, and 900 °C [35]. Copyright 2007, Elsevier. (**c**) Diagram of the change in response value of 5 wt% Al-doped TiO_2_ sensor with CO concentration at 700 °C, 800 °C, and 900 °C [35]. Copyright 2007, Elsevier. (**d**) Diagram of the change in response value of 7.5 wt% Al-doped TiO_2_ sensor with CO concentration at 700 °C, 800 °C, and 900 °C [35]. Copyright 2007, Elsevier. (**e**) A response diagram of the AZO nanoparticles as a function of CO concentration at the temperature of 300 °C [37]. Copyright 2014, Elsevier. (**f**) Double trimerization synthesis of cryptophane-A [40]. Copyright 2009, Elsevier. (**g**) Schematic diagram showing the process of creating FETs using thin films of PDPP4T-T-Pd(II) and PDPP4T-T-Hg(II) for the purpose of testing CO and H_2_S, respectively [43]. Copyright 2019, American Chemical Society.

Teleki et al. used flame spray pyrolysis (FSP) to prepare nanostructured anatase TiO_2_ for improving the performance of MEMS CO sensors [33]. Under the heat treatment of 900 °C, the conversion of anatase to rutile means the transformation of N-type to P-type sensing behavior. The original anatase sensor was unable to detect CO, while the rutile sensor could [33]. The operating temperature of the sensor is 500 °C, the detection range is 1–75 ppm, and the response time and recovery time are 30–60 s and 550 s, respectively [33]. The left axis of Figure 5a shows the signal change in the sensor exposed to 15–35 ppm CO after heat treatment at 900 °C [33]. It can be observed from the figure that in this range, the sensor signal decreases as the CO concentration increases [33].

#### 4.1.2. Metal/MOS

Choi et al. prepared nanopowders of Al-doped TiO_2_ ceramics by the citrate–nitrate automatic combustion method and used the powders to make MEMS CO sensors [35]. Figure 5b–d illustrates that upon the introduction of aluminum doping at concentrations of 0 wt%, 5 wt%, and 7.5 wt%, the sensor response is modulated by variations in CO concentration subsequent to the samples being subjected to calcination at temperatures of 700 °C, 800 °C, and 900 °C, respectively [35]. The resistance of TiO_2_ samples doped with Al is lower than that of pure TiO_2_, and the sensor response value reaches the maximum at 600 °C [35]. In addition, the sensor can also be used to detect O_2_ concentration [35]. Madler has fabricated a high-performance MEMS CO sensor with PT-doped SnO_2_ nanoparticles [36]. The gas-sensitive material is prepared by flame spray pyrolysis (FSP) and has a response value of 8 to 50 ppm CO in dry air at an operating temperature of 350 °C [36]. The sensor has high sensitivity, a low detection limit, and good reproducibility [36]. Changes in the thickness of the film affect the resistance of the sensor and can be used to improve the performance of the sensor [36]. Hjiri et al. developed an MEMS sensor using Al-doped ZnO (AZO) as a sensitive material using an improved sol–gel technique [37]. Since Al^3+^ has a smaller ion size compared to Zn^2+^, Al doping results in an increase in conductivity and a decrease in resistivity, significantly increasing the sensitivity of the sensor to CO gas [37]. According to the difference in Al doping content, the optimal operating temperature changes, and A3ZO obtains the best response at 300 °C [37]. In the detection range of low CO concentration (5–50 ppm), the response value shows an increasing trend with the increase in concentration (Figure 5e) [37]. When the temperature is 300 °C and the concentration is 50 ppm, the response reaches 80%, the sensitivity is 1.6, and the response time/recovery time is 7/30 s [37]. Anukunprasert et al. introduced a Ca/ZnO thin-film MEMS CO sensor coated with LGS [38]. The operating temperature is 400 °C, the operating frequency is 7.89 MHz, the detection range is wide (1000 ppm), and the tres and trec are 87 s and 132 s, respectively [38].

### 4.2. Carbon-Based Material

Tian et al. developed an MEMS CO sensor based on a two-dimensional (2D) plane of graphene with a large amount of gas adsorption at the active site [39]. In this paper, they studied different forms of graphene, including intrinsic graphene, S-W-defect graphene, and multi-vacancy-defect graphene, and used first principles based on density functional theory to judge the ability of this graphene to adsorb and detect CO [39]. The results show that the introduction of defects improves the sensitivity of graphene to CO and CO_2_, S-W defects make the sensitivity of the CO sensor reach 35.25%, and multi-vacancy defects make its sensitivity reach 4.14% [39].

### 4.3. Polymer Material

#### 4.3.1. Polymer

Bayram synthesized ferrocene branched-chain chitosan coating by the drip coating method and applied it to an MEMS CO sensor [40]. Because chitosan can enhance the sensing performance of CO at room temperature, a ferrocene-shell polycarbon alloy is a suitable material for making CO sensors [40]. The sensor works well at room temperature with a response time and recovery time of 38 s and 64 s, respectively [41]. The response of this CO sensor is linearly related to the gas concentration, and the detection range is 0–2000 ppm, and the response value increases as the carbon monoxide concentration increases [40]. The sensor is a quartz crystal microbalance (QCM) sensor, and its sensitivity is calculated using the Solbrey formula, Δf = 2f_0_^2^ Δm/A √ρ_q_u_q_. Δf and Δm represent the shift in quartz resonance frequency (Hz) and mass change (gm) related to surface adsorption on QCM. f_0_ is the fundamental frequency (Hz) of quartz; A denotes the active area (cm^2^) of the QCM rigid film on the electrode; ρ_q_ and u_q_ stand for the density (g/cm^3^) and shear modulus (Pa) of the piezoelectric quartz crystal, respectively. As depicted in Figure 5f, an electrochemical quartz crystal microbalance (EQCM) was employed to ascertain the shift in the resonant frequency of the QCM electrode [40]. By calculation, the sensitivity of the sensor Δf = 108.85 Hz/ppm [40]. Ping et al. reported a QCM gas sensor prepared by electrospray deposition of cryptonuclide A, which can be used to detect CH_4_, CO, and other gases [41]. When used as a CO sensor, it operates at 80 °C and has a sensitivity of 0.004 Hz/ppm [41].

#### 4.3.2. Metal/Polymer

Dixit et al. prepared metal halide-doped polyaniline (PANI) films by vacuum deposition for efficient and rapid detection of CO [42]. The sensor demonstrated its highest sensitivity to CO in different gas environments (R_a_/R_g_ = 800@150 ppm) [42]. Additionally, it showed response and recovery times of 5 s and 10 s, respectively, when exposed to CO [42]. Yang et al. incorporated a thymine group into the side chain of the diketopyrrole pyrrole (DPP)-based conjugated polymer PDPP4T-T to prepare MEMS gas sensors [43]. PDPP4T-T films have better crystallinity and higher charge mobility than similar polymers with pure alkyl chains [43]. Pd (II) or Hg (II) ions were incorporated into PDPP4T-T films by coordinating thymine with the ions at the air–water interface [43]. As shown in Figure 5g, the PDPP4T-T film and the Pd (II) or Hg (II) ions are transferred to the substrate by dropping a chloroform PDPP4T-T (0.1 mg/mL) solution onto the surface of an aqueous solution containing K_2_PdCl_4_ or Hg (ClO_4_)_2_, respectively [43]. PDPP4T-T thin-film field-effect transistors (FETs) containing Pd (II) ions are used to detect CO with a detection limit of 10 ppb, which has high sensitivity and selectivity for CO [43]. Moreover, PDPP4T thin-film FETs containing Hg (II) ions can be used to detect H_2_S with a detection limit as low as 1 ppb [43].

#### 4.3.3. Polymer/Polymer

Zhang et al. successfully synthesized poly (3,4-vinyldioxythiophene)/poly (styrene sulfonate)/polyethylpyrrolidone (PEDOT/PSS/PVP) composite nanofibers by electrospinning and used them to develop QCM CO sensors [44]. At room temperature, the resistivity of PEDOT/PSS/PVP nanofibers is 10~5 Ω·m. At low CO concentrations (5–50 ppm), the response of PEDOT/PSS nanofibers is linear with CO concentration [44]. When the CO concentration exceeds 50 ppm, the sensor’s adsorption capacity reaches saturation, and the resonant frequency range does not change [44].

## 5. Nitrogen Dioxide Sensors

Nitrogen dioxide, as a pollutant gas, often reacts violently with many organic compounds. It is one of the causes of ozone formation and plays an important role in the formation of acid rain. Therefore, the research and development of NO_2_ gas sensors is of great significance and can be applied to environmental protection, the manufacturing industry, scientific research, teaching, and other fields. As shown in Table 3, the MEMS NO_2_ sensors developed in recent years often have the advantages of excellent stability, fast response speed, high sensitivity, and good convenience. The following examples will be introduced.

### 5.1. MOS Material

#### 5.1.1. MOS

**Figure 6:**
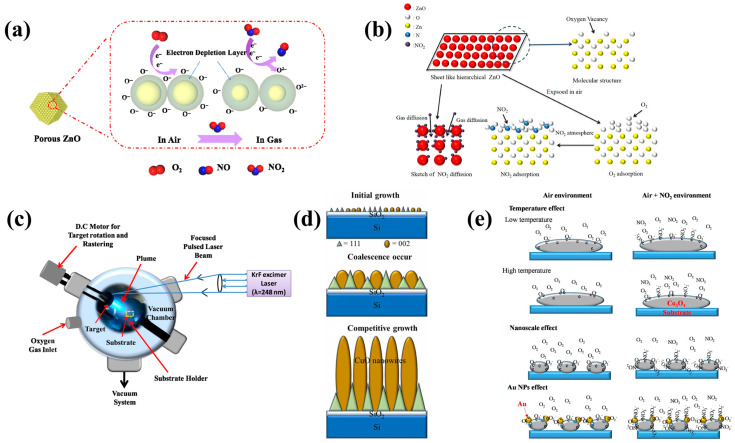
(**a**) Schematic diagram depicting the sensing mechanism of n-type ZnO-450 for NO_2_ detection [45]. Copyright 2024, Elsevier. (**b**) Schematic of the gas-sensing process of the MEMS NO_2_ sensor based on ZnO_1−x_ coating [47]. Copyright 2017, Journal of the Taiwan Institute of Chemical Engineers. (**c**) Schematic of pulsed laser deposition system used for deposition of PZT thin film [48]. Copyright 2018, Elsevier. (**d**) The initial growth of CuO-NWs is depicted in the schematic diagram, showing the occurrence of coalescence and competitive growth [49]. Copyright 2022, Elsevier. (**e**) The mechanism of Co_3_O_4_ NO_2_ gas sensing, taking into account the impact of temperature, nanoscale properties, and the presence of Au-NPs [50]. Copyright 2021, Elsevier.

Zhang et al. prepared ZnO-450, ZnO-600, and ZnO-750 layered porous zinc oxide materials with ZIF-90 that can promote gas transport and diffusion by the simple solution precipitation method using a zeolite imidazolate framework (ZIF-90) and the pyrolysis method at different temperatures [45]. They are applied as gas-sensitive materials for high-sensitivity MEMS NO_2_ sensors [45]. ZnO-450 is a typical n-type metal oxide semiconductor material. The schematic diagram of its gas-sensing mechanism is shown in Figure 6a [45]. When the ZnO-450 gas sensor is exposed to air, oxygen can be adsorbed on the surface of ZnO-450 to generate chemisorbent oxygen by trapping electrons in the ZnO-450 conduction band [45]. The electrons are reduced, and an electron depletion layer (EDL) is formed on the surface of ZnO-450 [45]. Then, when the ZnO-450 MEMS sensor is exposed to NO_2_, continues to capture electrons in the conduction of ZnO-450 and reacts with chemisorbent oxygen, resulting in a further increase in the thickness of the EDL on the ZnO-450 surface [45]. This sensor’s response to NO_2_ is higher than that of other gases, and the ZnO-450-based MEMS sensor shows better gas sensitivity at lower operating temperatures (190 °C) compared to ZnO-600 and ZnO-750 [45]. The response/recovery time is 9/26 s, and the resistance amplitude of the sensor gradually increases with the increase in NO_2_ concentration, showing its good reversibility [45]. As the concentration of NO_2_ increases in the range of 0.05–40 ppm, the response of the sensor shows an upward trend [45]. According to the experimental data, a high response value (242.18%@10 ppm) is obtained [45]. The LOD of NO_2_ ZnO-450 is 35 ppb based on the definition of LOD and the experimental results [45]. Geng et al. developed ZnO_1-x_ material with a hexagonal phase and a porous layered structure for the preparation of MEMS NO_2_ sensors [47]. They deposited a two-dimensional sheet of zinc oxide coating composed of nanoparticles in three steps on a flexible polypropylene paper equipped with gold electrodes [47]. The two-dimensional sheet coating composed of vinamil particles having a large surface–volume ratio and high porosity, which is conducive to the adsorption, desorption, and diffusion of gases (Figure 6b) [47]. The coating responds well to NO_2_ at room temperature (∆R/R_0_ = 2.61@1 ppm) [47]. Rana et al. reported a surface acoustic wave (SAW) resonator operating at 99.4 MHz using piezoelectric ST-cut quartz. The resonator device has been successfully integrated with the lead zirconate titanate (PZT) sensing layer and is highly selective to NO_2_ [48]. As indicated in Figure 6c, the process of depositing amorphous PZT films was executed utilizing a pulsed laser deposition (PLD) system [48]. The sensor device has a wide detection range (80–250 ppm) and high sensitivity (9.6 kHz/ppm) [48]. Hsueh et al. prepared CuO-NWs using RF sputtering technology for the preparation of MEMS NO_2_ gas sensors [49]. CuO-NWs grow vertically on the MEMS structure, in a process known as the competitive growth model [49]. As displayed in Figure 6d, the growth of CuO nanostructures on the substrate can be divided into three stages: initial growth, merging, and competitive growth [49]. In an environment with a NO_2_ concentration of 500 ppb and an operating temperature of 119 °C, the sensor has an average response of about 50.1% [49]. Studies have shown that CuO-NW/MEMS sensors have stability and repeatability for NO_2_ gas [49].

#### 5.1.2. Metal/MOS

**Figure 7:**
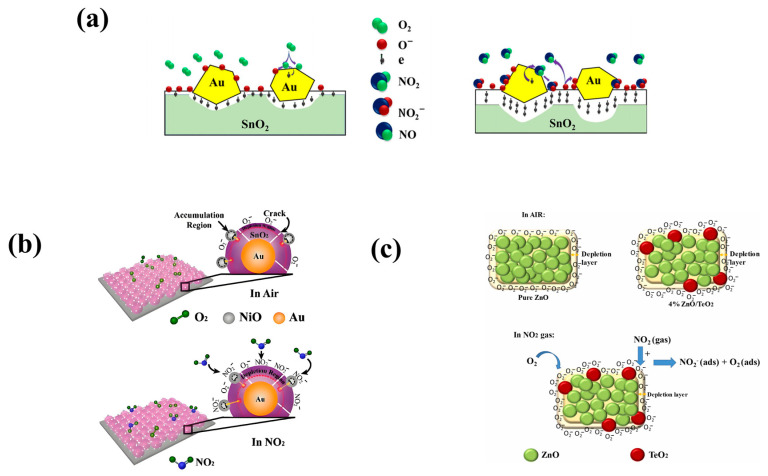
(**a**) Au/SnO_2_ sensor mechanism schematic diagram [51]. Copyright 2018, Elsevier. (**b**) Schematic model for the Au/SnO_2_/NiO sensor exposed in air and NO_2_ [52]. Copyright 2019, Elsevier. (**c**) Gas-sensing mechanism of TeO_2_/ZnO NO_2_ sensor [56]. Copyright 2024, Elsevier.

Hsueh et al. applied ultrasonic grinding technology to prepare Co_3_O_4_-NP materials with surface adsorption of Au-NPs with a diameter of about 1 nm to achieve a high sensitivity of MEMS NO_2_ sensors [50]. As shown in Figure 6e, this sensor makes it easier for NO_2_ molecules to react with Co_3_O_4_ surface electrons to improve sensitivity through temperature effect, nano-size effect, and Au-NP effect [50]. The response values of the sensor were measured at 100 ppb NO_2_ concentration and 114 °C, 136 °C, 157 °C, and 178 °C [50]. The sensor achieves a maximum response of 33% at an operating temperature of 136 °C [50]. The T_res_/T_rec_ of the sensor under this condition is 84 s/68 s, and the response is better than that of other MEMS gas sensors with Co_3_O_4_ as the sensitive material [50]. The sensing response of the Au/Co_3_O_4_-NP/MEMS sensor has a linear functional relationship with NO_2_ concentration, and the relationship between the two is exponential in the range of 10–10,000 ppb [50]. When the NO_2_ gas concentration is 10 ppm, the sensor response value is 225% [50]. Drmosh et al. reported a SnO_2_ film with highly uniformly dispersed Au nanoparticles on its surface [50]. The film was prepared by two-step annealing after sputtering and has high selectivity for NO_2_ [51]. It can be seen from Figure 7a that Au nanoparticles can be used as a catalyst leading to oxygen dissociation, which in turn promotes the adsorption of oxygen ions through the overflow process and improves the NO_2_ response characteristics of the sensor [51]. The response rate of the NO_2_ sensor at room temperature is 90%@50 ppm, which is 3.2 times and 5.5 times of the original SnO_2_ sensor and the SnO_2_ sensor with the Au layer, respectively, because the addition of highly dispersed Au nanoparticles significantly improves the light transmittance and crystallinity of the SnO_2_ film [51]. Wang et al. describe a heterogeneous Au/SnO_2_/NiO film for the preparation of MEMS NO_2_ sensing [52]. Figure 7b reveals the sensing mechanism of the sensor. When the Au/SnO_2_/NiO composite film is exposed to a NO_2_ environment, NO_2_ molecules react with surface chemisorbed oxygen to form adsorbed NO_2(ads)_ [52]. Because NO_2_ is much more electronegative than oxygen, NO_2_ can also extract electrons in both N-type SnO_2_ and P-type NiO [52]. The rapid decline in electron concentration in SnO_2_ leads to the expansion of the depletion region, which enables the detection of NO_2_ concentration. A method of preparing sensing materials by sputtering SnO_2_ was reported: sputtering SnO_2_ targets on self-assembled Au-NP arrays and then annealing them with H_2_ [52]. This method facilitates electron transfer in sensing materials, allowing MEMS-compatible heterogeneous fabrication methods to have wider applications in wafer-scale gases [52]. The sensor has a high response (185@5 ppm), high selectivity, and high stability with an LOD of 50 ppb due to the catalytic action of Au-NPs and the effective Schottky barrier and p-n junction formation [52].

#### 5.1.3. MOS/MOS

Jian et al. used an RF magnetron co-sputtering system to prepare Ta/In_2_O_3_ films with different Ta contents for the research and development of MEMS NO_2_ sensors [53]. The test results show that when the content of Ta is 2.33 at.%, the response and recovery time are relatively short, and the sensitivity is high [53]. At the same time, they also used a laser interferometric lithography system to model the performance of Ta/In_2_O_3_ rod arrays of different sizes [54]. The research results show that the number of rods and the surface-to-volume ratio increase with the decrease in the rod array period [54]. Using a sensor film with a 0.6 mm cycle Ta/In_2_O_3_ rod array, the NO_2_ gas sensor has a maximum response rate of 76.1, an optimal operating temperature of 110 °C, an LOD of 0.7 ppm, and a minimum response time and recovery time of 48 s and 329 s, respectively [54]. Wei et al. created a flexible MEMS NO_2_ sensor using amorphous rhodium oxide-decorated black indium oxide (RhO_x_/B-In_2_O_3_) as the sensing material [55]. The sensor exhibits excellent gas selectivity for NO_2_ at 5 ppm, high sensitivity (∆R/R_0_ = 42@5 ppm), and very short response/recovery time (8 s/17 s) at room temperature due to the presence of defects in the B-In_2_O_3_ material and the chemical sensitization and electron-rich properties of the amorphous RhO_x_ [55]. The research results show that RhO_x_/B-In_2_O_3_ composites have broad application potential in advanced gas sensing [55]. Yempati et al. developed MEMS NO_2_ sensors with TeO_2_-doped ZnO nanostructures by co-sputtering technology [56]. The sensor mechanism, which is mainly based on the formation of an electron depletion layer and the formation of a heterojunction between ZnO and TeO_2_, is shown in Figure 7c [56]. EDS mapping analysis confirmed that doped TeO_2_ may increase the base resistance value of the sensor. Compared with 2%ZnO-TeO_2_ and 4%ZnO-TeO_2_, the 8%ZnO-TeO_2_ material has the most uniform deposition on the entire surface, and the added TeO_2_ produces more absorption sites and more oxygen ions on the surface of the material. Therefore, the response to NO_2_ is more sensitive [56]. Testing revealed that the selectivity of the 4%TeO_2_-ZnO sensor for NO_2_ is much higher than that for other gases, and the T_res_/T_rec_ is 13/38 s [56]. At the optimum operating temperature of 100 °C, the response to NO_2_ at 200 ppb and to NO_2_ at 1 ppm reaches 42%, and the response degree increases with the increase in concentration in this interval [56]. The performance of TeO_2_-doped ZnO sensors is better than that of pure ZnO sensors [56].

### 5.2. Carbon-Based Material

**Figure 8:**
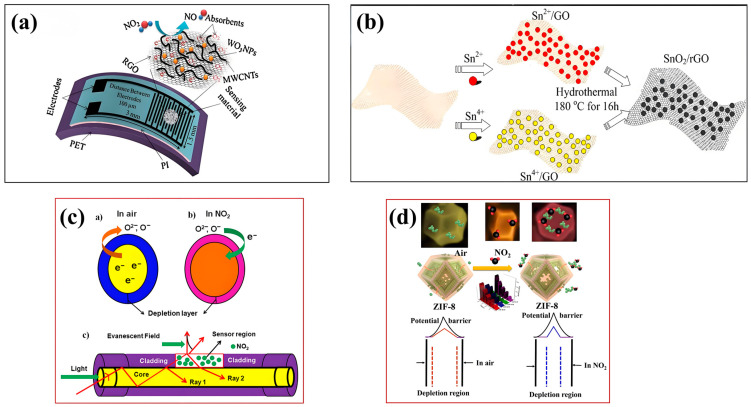
(**a**) The schematic illustration of the MEMS NO_2_ sensor based on WO_3_ NTS-MWCNT-RGO [61]. Copyright 2016, Sensors and Actuators B: Chemical. (**b**) Schematic representation of the synthesis process for SnO_2_/rGO nanocomposites using various tin salt precursors [63]. Copyright 2015, ACS Analytical Chemistry. (**c**) Schematic depiction of the NO_2_ sensing mechanism on the active surface area of MoS_2_/graphene [64]. Copyright 2020, Optics and Laser Technology. (**d**) Gas-sensing mechanism of ZIF-8 nanostructures [65]. Copyright 2021, Materials Research Bulletin.

Chung et al. fabricated a flexible MEMS NO_2_ sensor based on WO_3_ NTS-MWCNT-RGO mixed on a polyimide/polyethylene terephthalate (PI/PET) substrate [61]. The manufacturing procedure, as depicted in Figure 8a, entails the employment of photolithography and RF magnetron sputtering techniques to deposit a pair of aurous electrode fingers onto the PI/Si substrate [61]. Subsequently, the amalgamated droplets are meticulously positioned between these electrodes and subjected to drying on a heated plate [61]. Following this, the sample undergoes annealing treatment [61]. Ultimately, the PI tape is transferred onto the PET substrate [61]. The prepared sensor has good nitrogen dioxide sensing performance with a low LOD of 1 ppm and a sensitivity of 17%@5 ppm [61]. Even after a curvature angle of 90° and a bending/relaxation process are employed, the sensor showed no significant performance degradation. The research results show that the sensor has the advantages of high sensitivity, high mechanical flexibility, light weight, and economy [61]. Hua et al. investigated the response of SWNT-Fe_2_O_3_ composite films obtained by a simple annealing process to NO_2_, H_2_S, and other gases [62]. The uniform distribution of Fe_2_O_3_ nanoparticles in the porous film helps to improve the gas-sensitive properties of the material, while enabling it to sense more gases and eliminating conventional steps such as chemical functionalization or doping [62]. As a result, MEMS gas sensors prepared from this material can produce a stable response to H_2_S and exhibit an enhanced sensitivity to NO_2_ at room temperature (∆R/R_0_ = 18.3%@100 ppm) [62]. The film can be applied to sensors in configurations that directly fabricate large-area, flexible, or wearable films or fabrics [62]. Li et al. used a simple and cost-effective two-step hydrothermal and lyophilization strategy to prepare 3D SnO_2_/rGO composites with extremely large surface areas and stable nanostructures [63]. As shown in Figure 8b, two different tin salt precursors, Sn^2+^ and Sn^4+^, were utilized to form SnO_2_/rGO nanocomposites by hydrothermal reaction [63]. The gas sensor made of the composite material has high selectivity and good gas sensitivity to NO_2_ [63]. Detectable NO_2_ concentrations range from 2 ppm to 110 ppm with sensitivity up to R_a_/R_g_ = 11.8@110 ppm [63]. Sangeetha et al. reported an MEMS NO_2_ sensor with molybdenum disulfide (MoS_2_)/graphene material as the sensitive layer [64]. The combination of MoS_2_ spherical nanoparticles and two-dimensional graphene (2DG) sheets creates a large active surface area, enhancing the absorption properties of gas molecules in the presence of evanescent wave light [63]. As shown in Figure 8c, in this fiber optic sensor, 2D graphene sheets are interlinked with MoS_2_ nanoparticles, increasing the surface area and allowing gas molecules to interact through the presence of an evanescent field, thereby increasing the sensor output [64]. This sensor offers cost-effective production, high sensitivity (61%), and rapid response/recovery time (22 s/35 s), making it suitable for a wide range of applications [64].

Recently, some memristor-based ultra-sensitive gas sensors (gasistors) have been reported for the detection of NO_2_, NO, NH_3_, and other gases. HfO_2_ is often chosen as the material of memristors, and conductive filaments (CFs) are used to improve the gas sensitivity and accuracy of memristors [66]. Materials such as CNTs and MOS are used as sensitive materials for gasistors. The formation of CFs causes a transition of resistance states between high resistance (HRS) and low resistance (LRS), resulting in a local rearrangement of oxygen vacancies in high electric fields [66]. This gives the gasistor a gas-triggered switch and memory function [66]. The experimental results show that the addition of CFs can effectively solve the limitations of the CNT gas sensor in terms of sensitivity, recovery process, and humidity effect [58]. For instance, Chae et al. proposed a filament-based memristor heater (MH)-embedded transparent CNT gas sensor for the detection of NO_2_ gas at room temperature [58]. Nanoscale conductive filaments (CFs) are used to fabricate an MH based on the insulating material hafnium oxide (HfO_2_) [58]. The MH uses nanoscale CFs to apply heat directly below the sensing layer, allowing for lower power consumption and higher efficiency compared to conventional gas sensors [58]. The Joule heating in the MH solves the problem of CNTs having a large surface area and easily absorbing water and improving the humidity resistance of the material [58]. In addition, the microstructure changes (oxygen vacancies and gaps) caused by grain boundary formation during annealing reduce the operating voltage and power consumption of the MH [58]. As a result, the sensor has high transmittance and low power consumption (6.15 μW), little impact on humidity changes (<7.5%), and fast response/recovery (<1 ms/30.8 μW) [58]. Ahmad et al. report a conductive-filament-based heater (CFH)-embedded CNT NO_2_ sensor [59]. The pulse recovery mechanism is used to control the sensor recovery process within 1 ms [59]. The virtual heating of the application is increased, resulting in less degradation of the gas-sensing response characteristics at high relative humidity (RH) levels [59]. When vacuum heating at 0.5 V is applied, CFH-embedded CNT sensors degrade only 33% in the range of 30–90% RH levels. The sensor responds to ∆R/R_0_ = 52.20%@50 ppm [59].

### 5.3. Polymer Material

Navale et al. used PPy film as a sensing material and rotary coating technology to prepare NO_2_ sensors with excellent performance at room temperature [57]. The sensor has a detection range of 10–100 ppm, a response time of 126 s, and a sensitivity of (R_a_/R_g_ = 1.12@100 ppm) [57]. Gaikwad et al. deposited conductive polythiophene-modified SWNTs on Si/SiO_2_ substrate by a charge-controlled potentiostatic deposition method to prepare a high-performance MEMS NO_2_ sensor [60]. The performance of the sensor was tested by means of chemical resistance, and the test results showed that the sensitivity of the SWNT device to NO_2_ was enhanced by polythiophene modification. In addition, the sensor exhibits a very wide linear response range (0.01–10 ppm) [60].

### 5.4. MOF Material

Zhan et al. developed a polyhedral ZIF-8 nanostructured MOF material for the fabrication of MEMS NO_2_ sensors [65]. The gas-sensitive mechanism is shown in Figure 8d. The interaction between gas molecules and ZIF nanomaterials causes resistance changes [64]. The excellent gas-sensitive performance of the sensor is attributed to the free carrier density and high surface–volume ratio generated by the porous materials [64]. The sensor offers excellent gas-sensitive performance, including a wide detection range (10–100 ppm), high sensitivity (R_a_/R_g_ = 118.5@100 ppm), and fast response and recovery times (113.5 s and 111.5 s) [65]. The research results of this sensor material reflect the potential of MOF materials in the preparation of MEMS NO_2_ sensors and further expand the application range of MOF materials with high porosity in the gas sensor industrial environment [65].

## 6. Hydrogen Sulfide Sensors

Hydrogen sulfide is a toxic, acidic gas commonly produced in the refining of oil, the refining of natural gas, and catabolism in nature. Inhaling H_2_S at certain concentrations can cause damage to human cells. With the continuous contributions of researchers, a MEMS H_2_S sensor with high sensitivity, requiring minimal power, having a compact size, and possessing robust anti-interference capabilities and durability has been developed so that the H_2_S sensor can further play an important role in protecting safety. The performance of various MEMS H_2_S sensors is shown in Table 4.

### 6.1. MOS Material

#### 6.1.1. MOS

**Figure 9:**
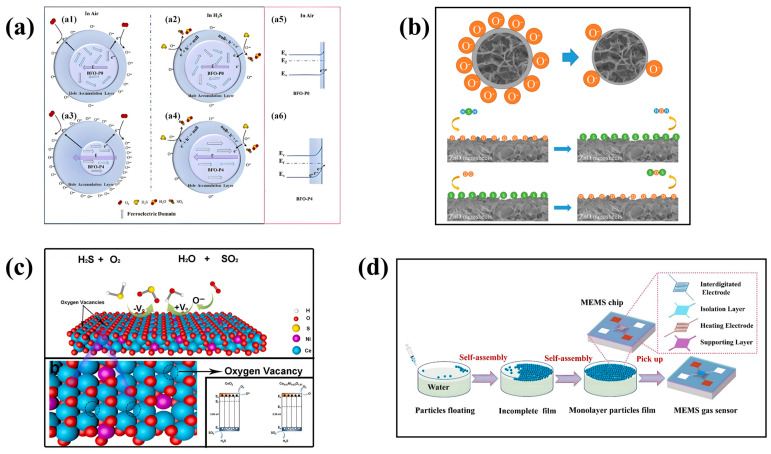
(**a**) Schematic diagram illustrating the sensing mechanism and the energy band structures of BFO-P0 and BFO-P4, (**a1**–**a4**) Schematic diagram illustrating of the sensing mechanism and (**a5**,**a6**) the energy band structures of BFO-P0 and BFO-P4. [68]. Copyright 2022, Elsevier. (**b**) Gas-sensing mechanism of ZnO sensor with H_2_S gas [69]. Copyright 2022, Elsevier. (**c**) Reaction model for Ce_0.97_Ni_0.03_O_1.97_ and schematic illustration for gas-sensing mechanism of CeO_2_ and Ce_0.97_Ni_0.03_O_1.97_ [72]. Copyright 2022, Elsevier. (**d**) The schematic diagram for the fabrication of a c/h-In_2_O_3_ MEMS gas sensor with monolayer particle film [75]. Copyright 2024, Elsevier.

Patricia et al. described the preparation and characterization of a flexible H_2_S sensor based on TiO_2_-NTs, using conventional microfabrication techniques to obtain an array of interleaving gold electrodes on one side and a universal heater on the back [67]. The sensor responds well at low temperatures with a sensitivity of ∆R/R_0_ = 144@38 ppm, enabling monitoring of H_2_S concentrations ranging from 6 ppm to 38 ppm at room temperature [67]. The research results show that the sensor has a promising application prospect in portable field detection based on low-cost nanomaterials [67]. Li et al. prepared an MEMS H_2_S sensor based on BiFeO_3_ (BFO) by a simple sol–gel method and treated ferroelectric semiconductor BFO nanoparticles by corona polarization, making the sensor still show excellent sensing performance for H_2_S at the ppb concentration level [68]. As can be viewed in Figure 9a, the excellent sensing performance of the BFO-P4 sensor on ppb H_2_S is mainly due to the adjustment of the electric polarization field by corona polarization, which leads to the difference in the hole accumulation layer thickness on the surface of BFO-P4 [68]. At the optimum operating temperature of 220 °C, the response of the BFO-P0, BFO-P2, and BFO-P4 sensors to 1.2 ppm H_2_S gas is very stable, and the fluctuation range is controlled within 2.6% [68]. Among them, the BFO-P4 sensor has good repeatability and has the smallest amplitude fluctuation (2.1%) in response to H_2_S [68]. At the same time, the BFO-P4 sensor can also effectively detect H_2_S of only 10 ppb, with a response value of R_g_/R_a_ = 1.03 [68]. Additionally, the sensor has been tested to have the ability to respond quickly (about 3 s) and recover (about 7 s) [68].

#### 6.1.2. Metal/MOS

Yempati et al. prepared zinc aluminum oxide (AZO) nanomaterials through hydrothermal synthesis and RF sputtering technology for the development of MEMS H_2_S sensors [69]. Figure 9b shows the chemisorption process of ZnO as an N-type semiconductor, which leads to the change in resistance [69]. When the best operating temperature of the sensor is 250 °C, the sensitivity ∆R/R_g_ = 14%@1000 ppb. Compared with CO, SO_2_, NO_2_, and other gases, the sensor has higher selectivity for H_2_S gas [69]. Dong et al. manufactured an MEMS H_2_S sensor based on Ni-doped CeO_2_ [72]. As shown in Figure 9c, doping with Ni creates more oxygen vacancies, and when hydrogen sulfide reacts with oxygen ions or is oxidized, electrons return to the cerium oxide surface, resulting in a thinner EDL and lower resistance of the sensor [72]. The change in Ce_0.97_Ni_0.03_O_1.97_ resistance results in a significant voltage change [72]. The response time and recovery time of the sensor are only 8 s and 13 s, respectively, and the LOD can be as low as 10 ppb [72]. The sensitivity can reach 3.1@500 ppb at the optimum operating temperature of 200 °C (in 20–90 RH% for 40 days) [72]. In addition, the sensor showed a lower LOD, down to 10 ppb H_2_S. The increase in oxygen vacancy and the catalytic action of hydrogen sulfide oxidation make the sensor more sensitive, selective, stable, moisture-proof, and safe [72].

#### 6.1.3. MOS/MOS

**Figure 10:**
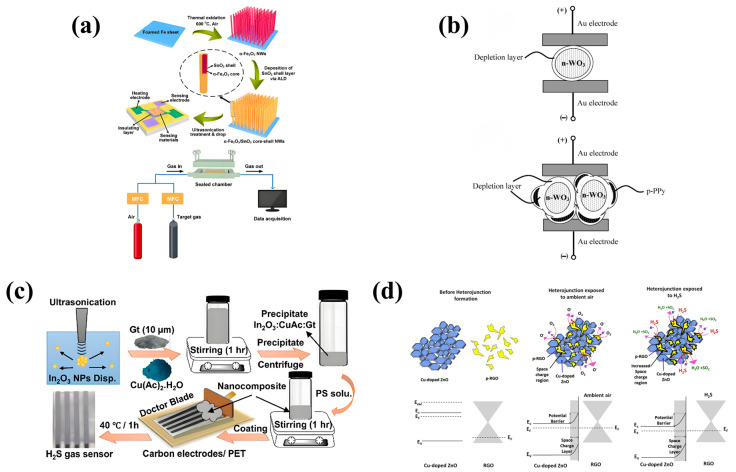
(**a**) The diagram illustrating the synthesis process of α-Fe_2_O_3_/SnO_2_ core–shell NWs on MEMS and the diagram depicting the gas-sensing measurement system [76]. Copyright 2024, Elsevier. (**b**) Comparable cross-sectional perspectives of H_2_S gas sensor utilizing WO_3_ film and PPy/WO_3_ nanocomposite film with a heterostructure (Au/p-PPy/n-WO_3_/p-PPy/Au) [83]. Copyright 2024, Elsevier. (**c**) Preparation procedure for the modified In_2_O_3_-NPs H_2_S sensors (MS) [86]. Copyright 2021, American Chemical Society. (**d**) Schematic representation of the gas-sensing mechanism and band diagram illustration of the Cu-doped ZnO/RGO nanocomposite sensor before and after exposure to ambient air and H_2_S gas, showing the heterojunction [87]. Copyright 2020, Elsevier.

As shown in Table 4, many H_2_S sensors use heterojunctions between MOS/MOS materials to improve sensor performance. Cho et al. developed MEMS H_2_S sensors with high sensitivity and low power consumption (<6 mW) using SnO_2_-ZnO hybrid nanostructures as materials [74]. Porous SnO_2_ films can be formed on the surface of ZnO-NWs by liquid phase deposition (LPD), and ZnO nuclei can be etched at the same time [74]. Finally, porous SnO_2_-NTS can be formed. Compared with ZnO-NWs, the sensing performance of the synthesized SnO_2_-NTs is significantly improved [74]. For example, at a H_2_S concentration of 20 ppm, the response R_a_/R_g_ of the ZnO-NWs sensor is 3.60, while the response R_a_/R of the SnO_2_-NTs sensor is 21.07 [74]. In addition, due to the heterojunction effect, the sensitivity (R_a_/R_g_ = 35.31@20 ppm) and response time (36–55 s) are further improved by forming SnO_2_-ZnO hybrid nanostructures [74]. Zhang et al. developed c/h-In_2_O_3_ with both rhombic corundum and cubic biphasic heterostructures [75]. A single-layer particle film MEMS gas sensor was obtained by the preparation process shown in Figure 9d [75]. First, the sensor is continuously aged in the air at 300 °C for 72 h; the target gas is dried and injected into the chamber through a syringe [75]. The gas concentration is determined according to the ratio of the injected gas volume to the chamber volume, and then the appropriate amount of liquid is extracted with a microsyringe [75]. It is then slowly injected into the hot plate of the gas chamber to form volatile gas and finally tested and calculated [75]. This metal oxide (MOS) material has abundant oxygen vacancies (61.1%) and more adsorbed oxygen (35.9%). Then, a MEMS gas sensor with a single layer of In_2_O_3_ particle film was prepared by self-assembly, and the abundant oxygen vacancies, two-phase heterojunction, and single-layer particle film are the reasons for the excellent H_2_S sensing performance of this c/h-In_2_O_3_ sensor [75]. As a metastable phase, h-In_2_O_3_ is easily converted into more stable c-In_2_O_3_, so the R_a_ value of the sensor always follows the trend of h-In_2_O_3_ < c-In_2_O_3_ < c/h-In_2_O_3_ [75]. The response value of the three sensors varies with the temperature, and the response rises continuously from 80 °C to 160 °C, reaching the best operating temperature [75]. The sensor has high sensitivity (54.4@50 ppm) and low LOD (20 ppb) [75]. H_2_S is diffused in the In_2_O_3_ particle layer in two different forms: interparticle diffusion and intraparticle pore diffusion [75]. The response time is positively correlated with the number of sensitive layers. When the number of layers changes from four to one, the response rate increases by 25 times, achieving an extremely fast response time (3.3 s) [75]. The excellent selectivity, remarkable repeatability, and long-term stability of the MEMS H_2_S gas sensor are attributed to the formation of a heterojunction between Fe_2_O_3_ and SnO_2_, the formation of a mat leading to an increase in chemisorbed oxygen, and the precise control of the tin oxide shell thickness and composition achieved by the ALD process [76]. The synthesis process principle and gas-sensing measurement system are shown in Figure 10a [76]. The α-Fe_2_O_3_/SnO_2_ core–shell NWs are ultrasonically dispersed into the deionized water of the foam iron sheet. The uniform dispersion is dripped onto the MEMS device and completely dried in the air [74]. An insulating layer is then inserted into the pair of gold sensing electrodes and a pair of platinum heating electrodes to prevent electrical crosstalk [76]. Finally, the MEMS gas sensor is prepared by connecting the MEMS device with the external circuit through a wire connection [76]. The JF02F gas-sensing measurement system was used to test the sensing characteristics of the sensor [76]. This study is based on the principle of the dynamic volumetric method, in which a dry standard target gas is dynamically mixed with high-purity air to obtain the desired concentration of the test gas [76]. Under the condition of a H_2_S concentration of 10 ppm and temperature of 250 °C, the sensor response reaches the peak [76]. α-Fe_2_O_3_/SnO_2_ core–shell nanowires with a shell thickness of 18 nm (F/S18) show the highest response of 4.3%, and T_res_/T_rec_ is 13.8/104.5 s [76].

### 6.2. Polymer Material

Su et al. utilized in situ photopolymerization to attach PPy and WO_3_ nanoparticles (PPy/WO_3_) to an Al_2_O_3_ substrate to prepare an MEMS H_2_S gas sensor at room temperature [83]. According to the equivalent profile diagram of the hydrogen sulfide gas sensor of the WO_3_ thin film and PPy/WO_3_ nanocomposite thin film (Figure 10b), it can be seen that the N-type semiconductor WO_3_ forms a p-n junction with the P-type semiconductor polypyrrole, whose depletion region is wider than that of WO_3_. Therefore, nanocomposite films are more sensitive to H_2_S than WO_3_ films [83]. The sensor based on the PPy/WO_3_ nanocomposite film responds ∆R/R_0_ to 81%@1 ppm at room temperature [83]. Microstructure observation showed that PPy was distributed in the PPy/WO_3_ nanocomposite membrane [83]. Therefore, a model of the barrier electron conduction of the composite material was used to determine the high response with a loss layer at the stretched PPy interface of WO_3_ thin films when detecting hydrogen sulfide gas adsorption at room temperature [83]. The sensor reacts well to very low concentrations of H_2_S gas at room temperature and is easy to manufacture [83]. Geng et al. prepared PPy and WO_3_ by chemical oxidation polymerization and emulsion methods, respectively, and prepared PPy/WO_3_ hybrid materials with different PPy mass percentages by mechanical mixing [84]. The proton doping process and N-type semiconductor effect make sensors based on the PPy/WO_3_ composites far more sensitive to H_2_S than those based on PPy or WO_3_ alone [84]. The recovery time and response time of the PPy/WO_3_ sensor to H_2_S are 70 s and 34 s, respectively, and its sensitivity is linear with H_2_S concentration, and the response to 1000 ppm H_2_S reaches ∆R/R_0_ = 60% [84].

### 6.3. Carbon-Based Material

Shboul et al. prepared a flexible H_2_S sensor using a nanocomposite mixture of In_2_O_3_, graphite (Gt) sheets, and polystyrene (PS) [85]. Nanocomposites composed of In_2_O_3_/10% Gt/17% PS are considered promising nanocomposites for the preparation of MEMS H_2_S sensors with excellent properties [85]. Both additives, Gt sheets and the PS modifier, help to increase the surface–volume ratio of the sensing film, improve the sensing performance of the In_2_O_3_ sensor and enhance its resistance to humidity changes [85]. Moreover, copper acetate (CuAc) is added to react with H_2_S gas to further improve the sensitivity and selectivity of the sensor to H_2_S [85]. It can be seen from Figure 10c that the improved In_2_O_3_ NP-based sensor MS2-50 keeps the gas chamber locked after each injection of H_2_S gas during the sensor test, showing good gas-sensitive performance [85]. The detection limit of this sensor is less than 100 ppb H_2_S, and its sensing performance is much better than that of some In_2_O_3_ H_2_S sensors, and its moisture resistance has been further improved (≈80% relative humidity) [86]. The device can be used to monitor the degradation of packaged foods, and the preparation method is a cost-effective disposable smart sensing technology [86]. Shewale et al. synthesized a low-temperature MEMS H_2_S sensor with Cu-doped ZnO (CZO)-decorated RGO nanostructures by the hydrothermal method [87]. As shown in Figure 10d, the formation of p-n heterojunctions between metal–semiconductor Schottky junctions and ZnO/RGO provides a large number of adsorption centers for hydrogen sulfide molecules, increasing the resistance of the sensor [87]. Due to the synergistic effect of Cu dopants and RGO, the performance of CZO/RGO sensors is enhanced compared to ZnO/RGO sensors [87]. The nanocomposite sensor has an inspection range of 136 ppb–250 ppm, a sensitivity of 0.87%@100 ppm at 24 °C, and a response time/recovery time of 14 s/32 s [87]. In addition to H_2_S, the sensor also shows high selectivity for H2 [87].

## 7. Ammonia Sensors

Ammonia is a pungent gas that can be produced by combining nitrogen and hydrogen, and it has the potential to cause irritation to the skin, eyes, and respiratory mucous membranes. Excessive inhalation will cause damage to the lungs, sensor, and eyes. MEMS technologies, including SAW technology, QCM technology, and so on, have been widely used in the research and development of NH_3_ sensors. QCM technology refers to the detection of gas by measuring the resonance frequency shift of quartz to detect the mass change in the sensing layer [4]. SAW technology refers to the excitation of gas monitoring by applying sine waves to a digital intersensor (IDT) deposited on a piezoelectric material [4]. The performance of devices using different technologies is shown in Table 5. The development of sensing films with different materials has further improved the stability and sensitivity of MEMS NH_3_ sensors.

### 7.1. MOS Material

#### 7.1.1. MOS

**Figure 11:**
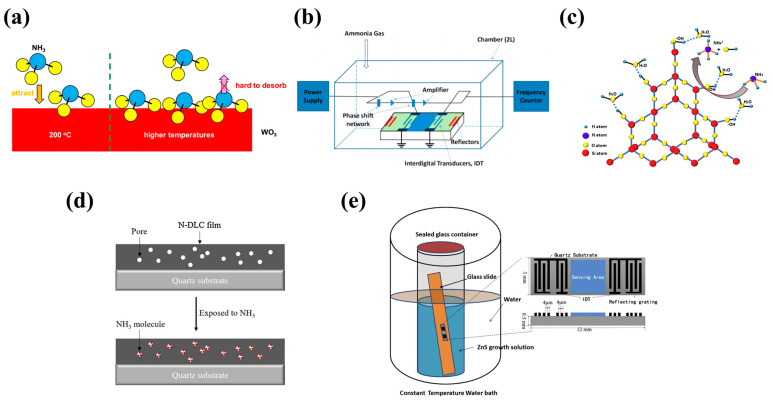
(**a**) Schematic operation of the WO_3_ thin-film MEMS NH_3_ sensor at temperatures exceeding 200 °C [91]. Copyright 2023, Elsevier. (**b**) Schematic diagram of an ammonia measurement system featuring a ZnO-nanorod MEMS sensor [96]. Copyright 2017, MDPI AG. (**c**) Schematic diagram of the reaction between NH_3_ and H_2_O molecules [98]. Copyright 2015, Elsevier. (**d**) Proposed mechanism for detecting NH_3_ using N-doped DLC film [102]. Copyright 2021, Elsevier. (**e**) Process diagram of preparing ZnS sensing layer on SAW device [108]. Copyright 2020, Elsevier.

Biskupski et al. prepared a TiO_2_ thin-film MEMS NH_3_ sensor using a new sol–gel-based hydrothermal process [88]. After annealing, the sensors were placed on a gas test bench with NH_3_ concentrations of 56 ppm, 103 ppm, and 156 ppm and a temperature of 350 °C for testing [88]. Anatase films have high selectivity for and sensitivity to NH_3_ [88]. Therefore, the sensor sensitivity R_a_/R_g_ reaches 1@1000 ppm [86]. Qiu et al. introduce a NH_3_ sensor based on TiO_2_-NWs gasistors [89]. The MOS-based sensor has integrated sensing and memory functions that traditional gas sensors do not have. At the same time, it has good sensing performance, T_res_/T_rec_ < 1 s, and sensitivity R_g_/R_a_ up to 164.2@1 ppm [89]. Wen et al. prepared WO_3_-NPs using ultrasonic grinding technology for the development of MEMS NH_3_ sensors [90]. The test results show that the sensitivity of the WO_3_-NPs gas sensor to NH_3_ is obviously better than that of CO, CO_2_, SO_2_, and other gases, the detection stability is good, and the sensing performance of NH_3_ is much better than that of the WO_3_ thin film and original WO_3_ powder sensor. When the operating temperature is 142 °C, the response R_a_/R_g_ of the sensor is 16% at 1.3 ppm [90]. Hsieh et al. used the anisotropic wet etching MEMS technique to fabricate a WO_3_ MEMS NH_3_ sensor with a suspension structure that can reduce the heat conduction of silicon [91]. As shown in Figure 11a, working at 200 °C, the sensor layer of WO_3_ is more able to attract NH_3_ molecules, and gas molecules can also desorb, resulting in the highest sensor response [91]. The chip size of the tiny ammonia sensor is only 5 mm × 5 mm. The sensitivity ∆R/R_0_ is up to 252%@5 ppm, with a response time of 30 s and an LOD of 40 ppb [91]. Quy et al. reported a wet chemical synthesis of an MEMS NH_3_ sensor based on ZnO nanorod-coated QCM [96]. Figure 11b shows that the SAW sensor was placed in a test chamber with a volume of 2000 mL. The dynamic volumetric method was used to inject gas into the laboratory with a syringe [93]. When the response reached an equilibrium state, the lid of the small chamber was removed, and the chamber was exposed to the atmosphere in the fume hood [96]. After the chamber was cleaned and the frequency of the sensor was stabilized, ammonia gas was injected into the test chamber again to complete the ammonia sensing characteristic test [96]. The selectivity of the sensor for NH_3_ is significantly higher than that of CO, CO_2_, NO_2_, liquefied petroleum gas (LPG), and other gases, the frequency change for 50 ppm NH_3_ at room temperature is about 9.1 Hz, and the response time and recovery time vary with the concentration of NH_3_ [96].

#### 7.1.2. MOS/MOS

Wang et al. fabricated ZnO/SiO_2_ (ZS) composite films using the sol–gel method and deposited them on the SAW resonator as the sensitive material for MEMS NH_3_ sensors [98]. The performance of ZnO:/SiO_2_ sensors in NH_3_ at different molar ratios (1:1, 1:2, and 1:3) was tested, and the results showed that the sensor response was best when the molar ratio was 1:2 [98]. The reaction between ammonia and water molecules is shown in Figure 11c [98]. Ammonia molecules can react with water molecules on the surface of SiO_2_ to initiate the proton conduction process through NH_4_^+^ [95]. This phenomenon leads to an increase in ionic conductivity, which is the reason for the good selectivity of the sensor [98]. This is attributed to the addition of SiO_2_, which enhances the absorption of NH_3_ by the film and the unique surface reaction on the composite film [98].

### 7.2. Carbon-Based Material

Li et al. created a new dual-layer film QCM NH_3_ sensor as a sensing layer [100]. The graphene oxide (GO) film acts as the isolating layer between the QCM electrode and the PANI sensing film [100]. Due to the high elastic modulus of the GO isolation layer, the quality factor of QCM is improved, the surface energy loss is reduced, and the prepared sensor has high stability and sensitivity (214 Hz/ppm) [100]. Zhu et al. reported a SAW NH_3_ gas sensor based on nitrogen-doped diamond-like (N-DLC) films prepared using microwave electron cyclotron resonance plasma chemical vapor deposition (ECR-PECVD) [102]. As shown in Figure 11d, the ammonia sensing mechanism of N-doped DLC films is proposed [102]. The pores in the film can trap ammonia molecules, resulting in a decrease in the porosity of the film [102]. By filling the pores in the sensitive film, the adsorbed ammonia gas molecules can lead to an increase in the area density and modulus of the N-DLC film, ultimately leading to the response caused by the elastic modulus [102]. The N-DLC films fabricated by ECR-PECVD are highly porous and physically and chemically selective in absorbing polar NH_3_ gas molecules [102]. The NH_3_ molecules adsorbed on the polar groups of N-DLC films lead to a decrease in their porosity, which leads to an increase in the elastic modulus of the films to detect NH_3_ [102]. Therefore, the concentration range of NH_3_ that can be monitored in situ by this sensor at room temperature is 10 ppb–100 ppm, and the response time/recovery time is only 5 s/29 s [102].

### 7.3. Polymer Material

Aditee et al. fabricated a PPy thin-film MEMS NH_3_ sensor through in situ chemical polymerization and employed Fourier transform infrared spectroscopy, scanning electron microscopy, and X-ray photoelectron spectroscopy for the analysis and characterization of the thin-film properties [103]. The test results show that the film is highly selective to NH_3_ at room temperature, the response value increases linearly with the increase in concentration in the detection range of 4–80 ppm, and the sensitivity ∆R/R_0_ can reach 16%@25 ppm [103]. Lin et al. fabricated a SAW gas sensor coated with Au/PPy films for monitoring low concentrations of ammonia [106]. Through the miniaturization of SAW chips and the use of polymers as sensing materials, the sensitivity of SAW gas sensors can be improved [106]. The SAW gas sensor is designed with a dual-device setup to minimize biases in detection, such as those caused by temperature and humidity [106]. The frequency offset of the SAW sensor is utilized for monitoring various concentrations of ammonia [106]. This developed SAW gas sensor demonstrates excellent repeatability and sensitivity, even when detecting low levels of NH_3_ [106]. The sensor has a sensitivity of 898 Hz/ppm and a detection range of 2–10 ppm [106].

### 7.4. Other Material

Shen et al. produced an MEMS sensor with good sensitivity, selectivity, repeatability, and reversibility for NH_3_ by depositing L-glutamate hydrochloride on a 128°YX LiNbO_3_ SAW delay line [107]. The sensor has a low LOD of 0.56 ppm at room temperature and a sensitivity of 74 Hz/ppm [107]. L-glutamate hydrochloride was degraded at a rate of only 0.01 ppm per day, indicating that the long-term stability of the sensing period could be guaranteed [107]. Since the cross-sensitivity of humidity interference is about 0.011, the effect of humidity on the device is negligible [107]. Long et al. synthesized ZnS mucosal nanostructures on ST-cut quartz SAW devices by chemical bath deposition to prepare MEMS NH_3_ sensors [108]. The preparation of the ZnS sensing layer on the SAW device is shown in Figure 11e [108]. The SAW unit is attached to a slide whose IDT and reflector are completely protected with polyimide tape [108]. It is then heated in a water bath in ZnS growth solution [108]. A thin film is then deposited in the sensing area, and the quartz SAW device is removed and soaked in ethanol [108]. Finally, it is ultrasonically cleaned with deionized water to remove any residue from the surface of the device [108]. Due to the larger specific surface area and more active sites of the ZnS mucosal nanostructure, the device has high sensitivity (−1.094 Hz/ppm) and high selectivity for NH_3_, with a response time/recovery time of 151 s/568 s [108]. Subramanian et al. utilized a surfactant-assisted solution combustion method to produce ultrafine Zn_3_(VO_4_)_2_ nanopowder, aiming for the advancement of fiber optic gas sensors [109]. The sensor operates at room temperature with a wide detection range (20–500 ppm), high sensitivity (0.019 μV ppm^−1^), and a response/recovery time of 46.8 min/59.0 min [109].

## 8. Conclusions and Prospect

MEMS gas sensors can detect H_2_, CO, NO_2_, H_2_S, NH_3_, acetone, formaldehyde, and other gases with their fast response, high sensitivity, small size, and easy integration, showing a good prospect of wide application and great commercial value. Materials such as MOS, MOF, CBM, SBM, and polymer materials, among others, form the sensors. These materials exhibit good sensing properties and can undergo doping, heterogeneous structure construction, and noble metal modification to enhance their performance. The previous article reveals that the exploration of MOF materials and SBMs is relatively less extensive than that of MOS materials. Each sensor type has a most suitable sensing material:

H_2_ Sensors: Pd/Pt-doped MOS materials showed superior performance. Hydrogen sensors made of these materials have excellent responses even to low concentrations of hydrogen. The addition of metal material changes the surface morphology and greatly improves the difficulty of hydrogen adsorption.

CO Sensors: Polymer and MOS materials demonstrated exceptional performance characteristics. So, researchers often choose them as gas-sensitive materials. There is less research on other kinds of materials, and more attention can be paid to CO sensors composed of metal materials and carbon materials in the future.

NO_2_ Sensors: MOS materials combined with CNTs or graphite showed remarkable performance. These materials have excellent performance with a fast response and low detection limit and can be widely used in industrial, chemical, and other fields.

H_2_S Sensors: Various MOS/MOS materials achieved better performance through the formation of p-n junctions. The change in electrical properties caused by heterojunction further improves the response sensitivity of H_2_S detection. However, these materials have poor moisture resistance. In recent years, some perovskite nanoparticle materials and hydrophobic molecular sieve materials have been used to prepare H_2_S sensors to improve their moisture resistance.

NH_3_ Sensors: MOS and MOFs were more commonly used because of their excellent gas sensitivity. MOS materials have been the mainstream choice for commercial NH_3_ sensors. While there have been significant advances in MOF-based gas sensor research, the development of MOFs for NH_3_ sensing is still in its infancy, and developing fast response sensors remains a challenge.

Although remarkable achievements have been made in scientific research and commercial use has been achieved, MEMS gas sensors still need to be improved. Some of the problems faced and their solutions, as well as development recommendations, are pointed out as follows:

(1) The existing gas sensors often have a high response temperature, poor response, and low sensitivity at low temperatures, which limits their application. Therefore, advanced nanomaterials and 2D materials need to be explored to ensure increased sensitivity and selectivity while reducing response temperature.
(2) Some metal oxide semiconductor materials and the noble metal materials used for modification are expensive, and their large-scale production is difficult to achieve. Appropriate MOS-based materials could be used for combination and preparation to reduce costs. In addition, it is necessary to pay attention to the development of low-cost manufacturing technology to further promote the industrialization of MEMS gas sensors.
(3) If the size of an MEMS device can be further reduced while its performance is improved, a wider range of applications in the chip can be achieved, while costs can be reduced and production can be expanded, which also requires the development of new devices and substrate materials.
(4) MEMS sensors can be integrated with IoT systems and intelligent technologies to achieve a wider range of applications. Some flexible MEMS can also be used to prepare wearable devices to achieve improved convenience.
(5) In order to achieve the purpose of environmental protection, energy-saving sensors using self-powered systems can be focused on in the future. In addition, more environmentally sustainable and biocompatible materials can be considered for the development of MEMS sensors.

In the future, more new materials will be discovered, and more technologies will be developed to integrate MEMS devices with portable devices, energy units, and smart homes with tiny sizes, high sensitivity, and multiple functions.

## Figures and Tables

**Table 3:** Performance comparison of nitrogen dioxide MEMS gas sensors and sensitive materials.

Sensitive Material Category	Sensitive Material	Fabrication Technique	Working Temperature (°C)	Detection Range (ppm)	Response Time (s)	Recovery Time (s)	Sensitivity	Ref.
MOS	ZnO-450	Pyrolysis of ZIF-90	190	0.035–40	9	26	2.42@10 ppm ^c^	[45]
MOS	ZnO	RF magnetron sputtering	Room temperature	0.4–16	N/a	N/a	6.0 kHz/ppm ^e^	[46]
MOS	ZnO_1-x_	Suspension flame spray (SFS)	Room temperature	0.25–1	180	300	2.61@1 ppm ^c^	[47]
MOS	PZT	Pulsed laser deposition	Room temperature	80–250	N/a	N/a	9.6 Hz/ppm ^e^	[48]
MOS	CuO-NWs	RF sputtering	119	100–500	136.3	272.3	50.1%@0.5 ppb ^c^	[49]
Metal/MOS	Au/Co_3_O_4_-NPs	Ultrasonic wave grinding technology	136	0.01–10	84	68	51%@0.2 ppm ^c^	[50]
Metal/MOS	Au-NP/SnO_2_ thin film	DC/RF sputtering	Room temperature	0.6–50	70	N/a	90%@50 ppm ^d^	[51]
Metal/MOS	Au-NP/SnO_2_/NiO thin films	E-beam evaporation	200	0.05–5	N/a	N/a	180%@5 ppm ^a^	[52]
Metal/MOS	Ta/In_2_O_3_	RF magnetron co-sputtering	120	1–100	59	339	64.5@100 ppm ^c^	[53]
Metal/MOS	Ta/In_2_O_3_	RF magnetron sputtering	110	0.7–100	48	329	76.1@100 ppm ^c^	[54]
MOS/MOS	RhO_x_/B-In_2_O_3_	Dip coating	25–125	1–20	8	13	42@5 ppm ^c^	[55]
MOS/MOS	TeO_2_/ZnO	Co-sputtering technique	100	0.2–1	13	38	80%@1 ppm ^d^	[56]
Polymer	PPy thin film	Spin coating technique	Room temperature	10–100	126	2170	1.12@100 ppm ^a^	[57]
CBM	CNT	RF sputtering	Room temperature	10	0.001	0.001	1.77@10 ppm ^a^	[58]
CBM	CNT	RF sputtering	Room temperature	10–50	N/a	N/a	52.20%@50 ppm ^c^	[59]
Polymer/CBM	Polythiophene-SWNTs	Potent ion static deposition	Room temperature	0.01–10	20	N/a	28@10 ppm ^b^	[60]
MOS/CBM	WO_3_-NP/MWCNT-RGO	Photolithography and radio frequency magnetron sputtering	Room temperature	1–25	420	800	17%@5 ppm ^c^	[61]
MOS/CBM	SWNT-Fe_2_O_3_	Floating catalytic chemical vapor deposition method	Room temperature	1–100	N/a	N/a	18.3%@100 ppm ^c^	[62]
MOS/CBM	SnO_2_/rGO	Hummers method	55	14–110	80	380	11.8@110 ppm ^a^	[63]
Inorganic substance/CBM	MoS_2_/Graphene	Chemical method	Room temperature	0–500	22	35	61%@500 ppm ^c^	[64]
MOF	Polyhedral ZIF-8 nanostructures	Solvothermal method	350	10–100	113.5	111.5	118.5@100 ppm ^a^	[65]

^a^ R = R_a_/R_g_. ^b^ R = R_g_/R_a_. ^c^ R = |R_g_ − R_a_|/R_a_ × 100%. ^d^ R = |R_g_ − R_a_|/R_g_ × 100%. ^e^ Δf = 2f_0_^2^ Δm/A √ρ_q_u_q_.

**Table 4:** Performance comparison of hydrogen sulfide MEMS gas sensors and sensitive materials.

Sensitive Material Category	Sensitive Material	Fabrication Technique	Working Temperature (°C)	Detection Range (ppm)	Response Time (s)	Recovery Time (s)	Sensitivity	Ref.
MOS	TiO_2_	Sputtering evaporation	70	6–38	N/a	N/a	144@38 ppm ^c^	[67]
MOS	BiFeO_3_	Facile sol–gel method	220	0.01–1.2	3	7	4.8@1.2 ppm ^a^	[68]
Metal/MOS	AZO	RF sputtering technique	250	0.2–1.0	N/a	N/a	14%@1000 ppb ^d^	[69]
Metal/MOS	Ag/ZnO	Co-sputtering technique	250	0.2–1.0	3	N/a	16%@0.001 ppm ^c^	[70]
Metal/MOS	Fe-NiO_x_ nanotubes	Drop coating	270	0.05–0.8	3.2	8.1	5.24@0.8 ppm ^c^	[71]
Metal/MOS	Ni/CeO	Drop coating	200	0.01–5.6	8	13	3.06@0.5 ppm ^a^	[72]
Metal/MOS	In_2_O_3_/Co_32_	Drop coating	185	0.05–2.5	30	76	N/a	[73]
MOS/MOS	ZnO/SnO_2_	LPD	25–300	1–20	36–55	N/a	35.31@20 ppm ^a^	[74]
MOS/MOS	c/h-In_2_O_3_	Self-assembly method	160	0.02–50	3.3	N/a	54.4@50 ppm ^a^	[75]
MOS/MOS	α-Fe_2_O_3_/SnO_2_	Atomic layer deposition (ALD)	250	1–10	13.8	104.5	4.3@10 ppm ^a^	[76]
MOS/MOS	Nb_2_O_5_/SnO_2_	ALD	275	1–20	20	97	4.0@20 ppm ^a^	[77]
MOS/MOS	CuO/SnO_2_	Dipping method	35	0.01–10	90	N/a	56,000@10 ppm ^a^	[78]
MOS/MOS	CuO/TiO_2_	Simple electrochemical anodization	Room temperature	3–400	41	92	46.81%@100 ppm ^c^	[79]
MOS/MOS	Cu_2_O/CuO	One-step reduction approach	95	0.05–1	N/a	76	2.1@0.05 ppm ^b^	[80]
MOS/MOS	CuO/WO_3_	RF sputtering technique	300	0–15	5	17 min	534@10 ppm ^a^	[81]
MOS/MOS	WO_3_-Bi_2_WO_6_	Facile hydrothermal technique	Room temperature	0.002–0.050	52	119	4.4@0.050 ppm ^a^	[82]
Polymer/MOS	PPy/WO_3_	In situ photopolymerization	Room temperature	0.1–1	360	12,600	81%@1 ppm ^c^	[83]
Polymer/MOS	PPy/WO_3_	Chemical oxidation polymerization and mechanical mixing	90	0.2–1	70	34	61%@1000 ppm ^c^	[84]
Polymer/MOS	PDPP4T-T-Hg(II)	Air–water interface coordination reactions of thymine groups with ions	Room temperature	0.001–1000	N/a	N/a	N/a	[44]
CBM/polymer/MOS	Gt/Ps/In_2_O_3_	Doctor blade method	Room temperature	0.1–1	N/a	N/a	70@1 ppm ^a^	[85]
CBM/polymer/MOS	Gt/Ps/CuAc/In_2_O_3_	Doctor blade method	Room temperature	0.1–3	60	N/a	18.1@0.1 ppm ^a^	[86]
Metal/MOS/CBM	Cu/ZnO/RGO	RF magnetron sputtering	24	0.136–250	14	32	0.87%@100 ppm ^c^	[87]

^a^ R = R_a_/R_g_. ^b^ R = R_g_/R_a_. ^c^ R = |R_g_ − R_a_|/R_a_ × 100%. ^d^ R = |R_g_ − R_a_|/R_g_ × 100%.

**Table 5:** Performance comparison of NH_3_ MEMS gas sensors and sensitive materials.

Sensitive Material Category	Sensitive Material	Fabrication Technique	Working Temperature (°C)	Detection Range (ppm)	Response Time (s)	Recovery Time (s)	Sensitivity	Ref.
MOS	TiO_2_	Hydrothermal process	350	56, 103, 156	N/a	N/a	1@1000 ppm ^a^	[88]
MOS	TiO_2_	Hydrothermal method	Room temperature	1	<1 s	<1 s	164.2@1 ppm ^b^	[89]
MOS	WO_3_	Ultrasonic wave grinding	200	0.04–5	30	135	2.525@5 ppm ^a^	[90]
MOS	WO_3_-NPs	Plasma-enhanced chemical vapor deposition (PECVD)	142	1.3	59	47	16%@1.3 ppm ^c^	[91]
MOS	Bismuth tungstate (Bi_2_WO_6_) nanomaterials	Hydrothermal technique	Room temperature	0–500	N/a	N/a	5 counts/kpa	[92]
MOS	ZnO-NWs	Drop-coating technique	Room temperature	1000	4–5	N/a	−956 Hz/ppm ^d^	[93]
MOS	ZnO nanorods	Wet chemical route	Room temperature	150	N/a	N/a	0.62 Hz/ppm ^d^	[90]
MOS	ZnO-NRs	Hydrothermal method	Room temperature	800	720	1400	11.33@100 ppm ^a^	[94]
MOS	ZnO-NRs	Hydrothermal method	Room temperature	100	151	568	−1.094 Hz/ppm ^d^	[95]
MOS	ZnO nanofilm	Sol–gel and spin coating	Room temperature	100	143	426	−0.307 Hz/ppm ^d^	[96]
MOS	Three-dimensional ZnO nanoflowers	Drop coating	Room temperature	0–7281	26	36	4.32 counts ppm^−1^	[97]
MOS/MOS	ZnO/SiO_2_(ZcS) composite film	Sol–gel method and spin coating	Room temperature	50	N/a	N/a	0.02264 kHz/ppm ^d^	[98]
CBM	SWNT film	CVD	Room temperature	10–200	250	300	4%@200 ppm ^c^	[62]
CBM	Polyaniline/graphene	Drop-coating method	Room temperature	20–100	50	35	85 Hz/ppm ^d^	[99]
CBM	Polyaniline/GO layer	Drop-coating method	Room temperature	800	79	3	214 Hz/ppm ^d^	[100]
CBM	CA/PEI/GO nanofiber	Sol–gel method	Room temperature	80	<10	N/a	11.3 Hz/ppm ^d^	[101]
CBM	N-DLC	ECR-PECVD	Room temperature	0.1–100	5	29	3.3 kHz/ppm ^d^	[102]
Polymer	PPy thin film	Polymerization process	Room temperature	4–80	20	800	16%@25 ppm ^c^	[103]
Polymer	PPy nanofiber	Reactive template approach	Room temperature	20–150	15	N/a	1.53@20 ppm ^b^	[104]
Polymer	Iodine-doped polythiophene (PTh) film (IPTF)	Electrophoretic deposition technique	Room temperature	460–1850	78	346	94.64%@1850ppm ^c^	[105]
Metal/polymer	Au/PPy	Spin coating	Room temperature	2–10	59	72	898 Hz/ppm ^d^	[106]
Inorganic substance	L-glutamic acid hydrochloride	Air-brushed coating	Room temperature	0.56–4.0	<300	<180	74 Hz/ppm ^d^	[107]
Inorganic substance	ZnS nanostructures	Chemical bath deposition method	Room temperature	20	45	148	62.5 Hz/ppm ^d^	[108]
Inorganic substance	Zinc vanadate (Zn_3_(VO_4_)_2_) nanopowder	Dip coating	Room temperature	20–500	2808	3540	0.019 µV/ppm	[109]

^a^ R = R_a_/R_g_. ^b^ R = R_g_/R_a_. ^c^ R = |R_g_ − R_a_|/R_a_ × 100%. ^d^ Δf = 2f_0_^2^ Δm/A √ρ_q_μ_q_.
